# Aberrant expression of the COX2/PGE_2_ axis is induced by activation of the RAF/MEK/ERK pathway in BRAF^V595E^ canine urothelial carcinoma

**DOI:** 10.1038/s41598-020-64832-5

**Published:** 2020-05-08

**Authors:** Ryohei Yoshitake, Kohei Saeki, Shotaro Eto, Masahiro Shinada, Rei Nakano, Hiroshi Sugiya, Yoshifumi Endo, Naoki Fujita, Ryohei Nishimura, Takayuki Nakagawa

**Affiliations:** 1 Laboratory of Veterinary Surgery, Graduate School of Agricultural and Life Sciences, University of Tokyo, 1-1-1, Yayoi, Bunkyo-ku, Tokyo, 113-8657 Japan; 2 Laboratory of Veterinary Biochemistry, Department of Veterinary Medicine, Nihon University College of Bioresource Sciences, 1866 Kameino, Fujisawa, Kanagawa 252-0880 Japan

**Keywords:** Bladder cancer, Bladder cancer

## Abstract

Cancer-promoting inflammation is an important event in cancer development. Canine urothelial carcinoma (cUC) overexpresses prostaglandin E_2_ (PGE_2_) and has a unique sensitivity to cyclooxygenase 2 (COX2)-inhibiting therapy. In addition, majority of cUC harbour *BRAF*^*V595E*^ mutation. However, mechanisms underlying aberrant PGE_2_ production in *BRAF*^*V595E*^ cUC patients remain unclear. Drug screening revealed that inhibition of RAF/MEK/ERK pathway, p38 and JNK pathway reduced PGE_2_ production in cUC cells. By pharmacological inhibition of the multiple components in the pathway, activation of the ERK MAPK pathway was shown to mediate overexpression of COX2 and production of PGE_2_ in *BRAF*^*V595E*^ cUC cells. *In silico* gain-of-function analysis of the *BRAF* mutation also implicated involvement of mutation in the process. The positive association between ERK activation and COX2 expression was further validated in the clinical patients. Moreover, it was also suggested that p38 and JNK regulates PGE_2_ production independently of ERK pathway, possibly through COX2-dependent and COX1-/COX2- independent manner, respectively. In conclusion, this study demonstrated that activation of ERK induces production of PGE_2_ in *BRAF*^*V595E*^ cUC cells, which is also independently regulated by p38 and JNK. With its unique vulnerability to COX-targeted therapy, *BRAF*^*V595E*^ cUC may serve as a valuable model to study the tumour-promoting inflammation.

## Introduction

Inflammation occurring in cancer tissues, referred to as cancer-promoting inflammation, promotes cancer progression by providing various factors, such as growth factors, pro-angiogenic factors, enzymes required for cancer invasion and metastasis, and immune-suppressive factors^1,2^. Being recognised as one of the hallmarks of cancer, cancer-promoting inflammation is a promising target for cancer therapy^1^. Among various processes causing cancer-promoting inflammation, the pathway of cyclooxygenase 2 (COX2) and its metabolite prostaglandin E_2_ (PGE_2_) has been widely accepted to be important in human cancers^2^. COXs are rate-limiting enzymes required for the biosynthesis of PGs in the arachidonic acid cascade, and PGE_2_ is the most abundant COX metabolite. Under physiological conditions, COX2 and PGE_2_ are induced during inflammatory processes and act as pro-inflammatory factors^3^. COX2 overexpression and subsequent PGE_2_ overproduction are observed in various human cancers^4–7^ and play a crucial role in the development of the tumour-promoting inflammatory microenvironment^2,8^.

Canine urothelial carcinoma (cUC) is the most common malignancy affecting the lower urinary tract of dogs. cUC is a unique tumour often well managed using COX inhibitors or non-steroidal anti-inflammatory drugs (NSAIDs)^9^. In addition, COX2 is overexpressed in cUC^10–12^. We previously reported that cUC cell lines overexpress PGE_2_ *in vitro* compared to other canine tumour cell lines with different tissues of origin^13^. Further, we suggested that aberrant PGE_2_ production is important for the development of tumour microenvironment and not for cell proliferation or survival^13^. However, the pathway that induces upregulation of COX2/PGE_2_ axis in cUC cells was not elucidated.

Another characteristic of cUC is that a single nucleotide mutation in the *BRAF* gene, V595E, is detected in 70%–80% of canine patients^14,15^. BRAF is an isoform of RAF serine/threonine kinase, which belongs to the RAF/MEK/ERK mitogen-activated protein kinase (MAPK) pathway. This pathway is one of the most important signalling pathways that transmit extracellular signals to cell nuclei, thereby regulating cell proliferation, differentiation, survival and various other cellular functions. The human counterpart of this mutation, which is recognised as *BRAF*^*V600E*^, is frequently observed in a variety of human malignancies such as malignant melanoma, colorectal cancer and papillary thyroid cancer^16–18^. The *BRAF*^*V600E*^ mutation reportedly induces oncogenic cellular proliferation via constitutive activation of the ERK MAPK pathway^16,19^. Therefore, several molecular targeting drugs against *BRAF*^*V600E*^ have been established and have improved the prognosis of patients with cancer^20,21^. Although canine *BRAF*^*V595E*^ is also suggested to contribute to constitutive activation of the ERK MAPK signalling cascade, its importance in cUC progression remains unclear.

In this study, we screened molecular targeting agents to determine the pathways involved in PGE_2_ production in a *BRAF* mutant cUC cell line. We investigated the contribution of the ERK MAPK pathway in the regulation of the COX2/PGE_2_ axis including various cUC cell line, most of which harboured *BRAF*^*V595E*^ mutation. Next, we investigated the relationship between *BRAF* genotype, ERK phosphorylation and COX2 expression in cUC tissues. Eventually, involvement of the other two MAPK pathways has been also evaluated. Our findings indicate a novel association between the activation of the ERK MAPK pathway in *BRAF* mutant cUC cells and dysregulation of the COX2/PGE_2_ axis.

## Results

### *In vitro* drug screening for disruption of PGE_2_ production in BRAF mutant cUC cells

**Figure 1:**
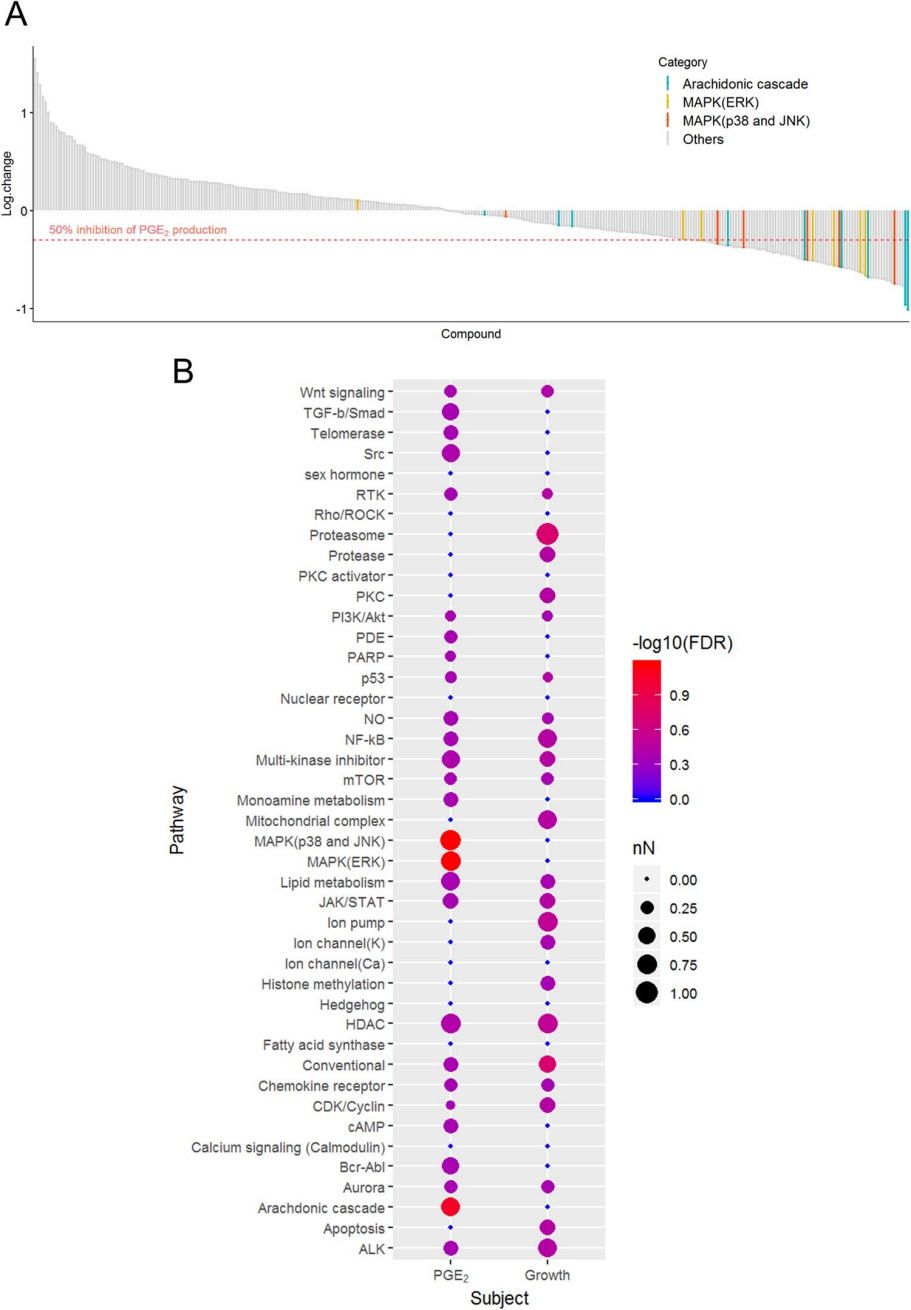
PGE_2_ production in cUC cells (Sora) during drug screening. (**A**) Y axis represents log 10 values of changes in PGE_2_ production for each inhibitor (n = 331). The drugs which belong to selected pathways are coloured as indicated. (**B**) Visualisation and comparison of the results from drug screening for PGE_2_ production and cell growth^52^. Drugs which showed ≥50% reduction in PGE_2_ production and cell density were included in statistical analysis. Categories which have more than 3 inhibitors are presented in this graph. Node size represents proportion of the drugs which showed reduction more than the threshold in each category. Colour represents statistical significance.

We previously reported that cUC cell lines overexpress PGE_2_^13^. To elucidate the mechanisms underlying aberrant PGE_2_ production in cUC cells, we screened 331 inhibitor compounds using SCADS inhibitor kit 1–4 obtained from Molecular Profiling Committee, Grant-in-Aid for Scientific Research on Innovative Areas “Advanced Animal Model Support (AdAMS)” from The Ministry of Education, Culture, Sports, Science and Technology, Japan (KAKENHI 16H06276; see Supplementary Table S1). A *BRAF* mutant cUC cell line, Sora, was treated with each inhibitor compound at 10 µM for 12 h. A concentration of 10 µM was used during the screening process according to manufacturer instructions in consideration of the IC_50_ value of each reagent to inhibit its target molecule(s). The amount of PGE_2_ in the medium was quantified after the treatment, and percent change in PGE_2_ production with respect to that in vehicle control (DMSO) was calculated (Fig. 1A and see Supplementary Table S1). Eighty compounds showed ≥ 50% reduction in PGE_2_ production in the cUC cells. After categorisation of all the compounds into their specific targeting biological pathways, enrichment of each category for the PGE_2_-suppressing compounds was analysed. Statistical analysis revealed that compounds targeting the arachidonic acid cascade (FDR = 0.086), ERK MAPK pathway (FDR = 0.067) and p38 and JNK MAPK pathways (FDR = 0.067) were enriched in these 80 compounds (Table 1 and Fig. 1A,B). In addition, the compounds against the enriched pathways did not show strong cytotoxic effects on *BRAF* mutant cUC cells (Fig. 1B and Supplementary Fig. S1). Since the arachidonic acid cascade falls directly upstream of PGE_2_ production, it was considered that the inhibitory effect observed in the screening does not explain mechanisms for the induction of COX2/PGE_2_ in cUC cells. As the cell line harboured *BRAF* mutation, we initially focused on a role of the ERK MAPK pathway in the regulation of COX2/PGE_2_. Later on, the investigation was extended to p38 and JNK MAPK pathways.

**Table 1:** Percent change in PGE_2_ by the treatment of the inhibitors targeting Arachidonic acid cascade, ERK MAPK pathway, and p38 and JNK MAPK pathway.

Pathway	Target	Compound	Percent change (%)
Arachidonic acid cascade (FDR = 0.086)	COX-1	Sulindac sulphide	−89.1
	COX-1	Valeryl salicylate	−55.4
	COX-2	NS-398	−90.2
	COX	Sodium salicylate	−8.7
	PLA2	cPLA2inhibitor	−78.9
	PLA2	OBAA	−29.9
	lipoxygenase	Nordihydroguaiaretic acid (NDGA)	−28.7
	12, 15-lipoxygenase	ETYA	−73.4
	12-lipoxygenase	Baicalein	−68.0
MAPK (ERK) pathway (FDR = 0.067)	MEK	PD 98059	−76.3
	MEK	U0126	−69.1
	MAPK	ERK inhibitor II	−50.0
	MEK	MEK inhibitor I	−78.2
	Raf	RAF1 kinase inhibitor I	−72.5
	Raf	ZM 336372	−48.2
	Tpl2	Tpl2 kinase inhibitor	26.2
	BRAF	Vemurafenib	−69.1
MAPK (p38 and JNK) pathway (FDR = 0.067)	JNK	SP600125	−53.9
	p38 (MAPK)	PD169316	−82.0
	p38 (MAPK)	SB 203580	−73.0
	JNK	JNK inhibitor VIII	−12.4
	p38	SB202190	−68.3
	p38	SB239063	−57.6

### Effects of ERK MAPK inhibition on the expression of the COX2/PGE_2_ axis

**Figure 2:**
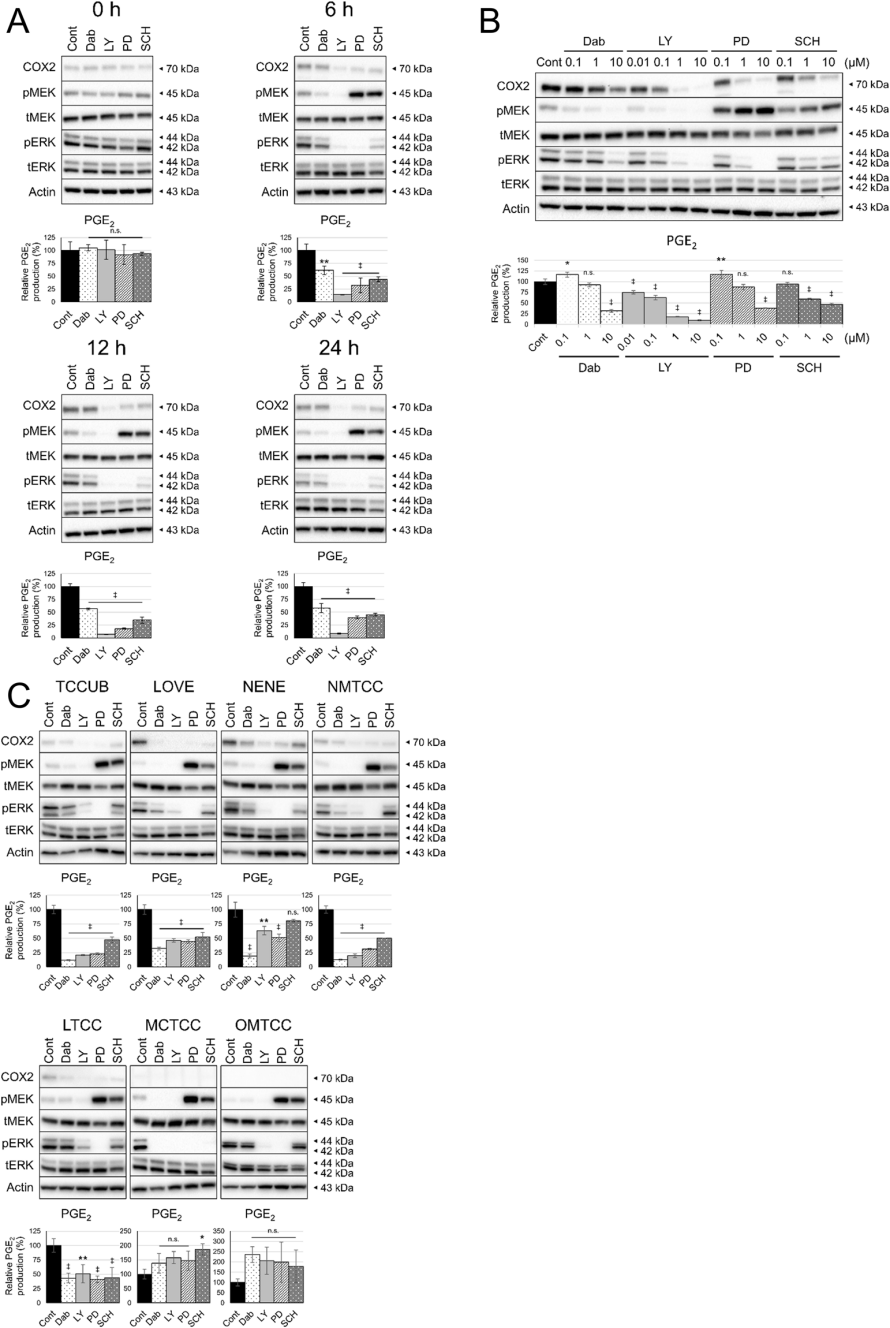
Effect of ERK MAPK inhibition on COX2 expression and PGE_2_ production. Protein levels in whole cell lysates were detected by Western blotting with Actin as loading control. Amount of PGE_2_ in culture medium was measured by enzyme-linked immunosorbent assay and normalised to cell number. Bar graph represents % control of PGE_2_ production. (**A**) cUC cells (Sora) were treated with vehicle (dimethyl sulfoxide; Cont) and inhibitors of BRAF (Dabrafenib; Dab), pan-RAF (LY3009120; LY), MEK (PD0325901; PD) and ERK (SCH772984; SCH) at 1 μM for indicated time. (**B**) cUC cells (Sora) were treated with vehicle (Cont), Dabrafenib (Dab), LY3009120 (LY), PD0325901 (PD) and SCH772984 (SCH) for 12 h at indicated dose. (**C**) cUC cell lines (TCCUB, Love, Nene, NMTCC, LTCC, MCTCC and OMTCC) were treated with vehicle (Cont), Dabrafenib (Dab) at 10 μM, LY3009120 (LY) at 1 μM, PD0325901 (PD) at 10 μM and SCH772984 (SCH) at 10 μM for 12 h. Data are presented as mean ± SD of three experiments. *Indicates p < 0.05, **p < 0.01, ^‡^p < 0.001 compared to vehicle control (Dunnett’s test).

The inhibitors, aim at various components of the ERK MAPK pathway, were used for the experiments; dabrafenib (Dab) (BRAF inhibitor), LY3009120 (LY) (pan-RAF inhibitor), PD0325901 (PD) (MEK inhibitor) and SCH772984 (SCH) (ERK inhibitor). All compounds were used at 1 µM for 0, 6, 12 and 24 h. After 6 h of treatment, it was noted that COX2 expression and PGE_2_ production were decreased by ERK MAPK inhibitors accompanying dephosphorylation of ERK (Fig. 2A). Next, cells were exposed to each compound at varying concentrations for 12 h. It was confirmed that ERK MAPK inhibitors suppressed COX2 expression and PGE_2_ production in cUC cells in a dose-dependent manner (Fig. 2B). In the both experiments, RAF inhibitors (Dab and LY) induced dephosphorylation of MEK and ERK while MEK and ERK inhibitors (PD and SCH) caused overphosphorylation of MEK, which could be attributed to the release from the negative feedback loop of the pathway^22^. PD is a non-ATP-competitive inhibitor which inhibits phosphorylation of ERK by MEK.

To generalise the finding that inhibition of ERK MAPK pathway leads to suppression of COX2/PGE_2_ in cUC cells, another six *BRAF* mutant cUC cell lines (TCCUB, Love, Nene, NMTCC, LTCC and MCTCC) and one *BRAF* wild type cUC cell line (OMTCC) were further included in the study. The characteristics of the cell lines at basal state has been evaluated in advance (Supplementary Fig. S2A and B). Four of eight cUC cell lines (Sora, TCCUB, Nene, NMTCC) showed increased PGE_2_ production as we have published previously^13^. Two (Love, LTCC) showed moderate and the other two (MCTCC, OMTCC) did low production of PGE_2_. Phosphorylation of ERK was observed in all the cell line including *BRAF*^*WT*^ OMTCC to some extent. Activation of p38 and JNK pathways and expression of COX1 were also observed in several cell lines.

These additional seven cell lines were treated with the ERK MAPK inhibitors with the varying doses for 12 h. In five cell lines (TCCUB, Love, Nene, NMTCC, LTCC), which showed comparatively higher PGE_2_ production at basal state (see Supplementary Fig. S2B), decrease in COX2 expression and PGE_2_ production was observed when the various components of ERK MAPK pathway was inhibited (Fig. 2C). It is noted that this effect was not observed in the low PGE_2_-producing cell lines (OMTCC and MCTCC) although the drugs successfully inhibited phosphorylation of ERK (Fig. 2C). One reason for this observation may be that these two cell lines do not express COX2 at basal state (Fig. 2C and Supplementary Fig. S2A). Collectively, it can be said that inhibition of ERK MAPK leads to decrease in COX2 expression in the *BRAF* mutant cUC cell lines.

We further explored the association between *BRAF*^*V595E*^ gain-of-function mutation and expression of COX2. Public database (PubMed and GREIN) search was conducted and data from the four literature were accumulated, in which transfection of *BRAF*^*V600E*^ into human normal cells was performed and changes in the transcriptome were evaluated^23–27^. The information of the retrieved data was summarised in Supplementary Table S2. By comparing the fold changes of normalised COX2 (*PTGS*2) expression, it was revealed that forced expression of *BRAF*^*V600E*^ in human normal cells frequently induced strong transcription of *PTGS2* in more than one type of cell (Supplementary Fig. S3A). Conversely, there was no clear trend regarding COX1 (*PTGS1*) expression (Supplementary Fig. S3B). This result suggests a hypothesis that *BRAF*^*V595E*^ mutation is a direct cause of COX2 overexpression and subsequent PGE_2_ overproduction in cUC cells, which needs to be further examined by future transfection and loss of function studies using canine cell lines.

### Association between BRAF mutation, ERK phosphorylation and COX2 expression in cUC patients

**Figure 3:**
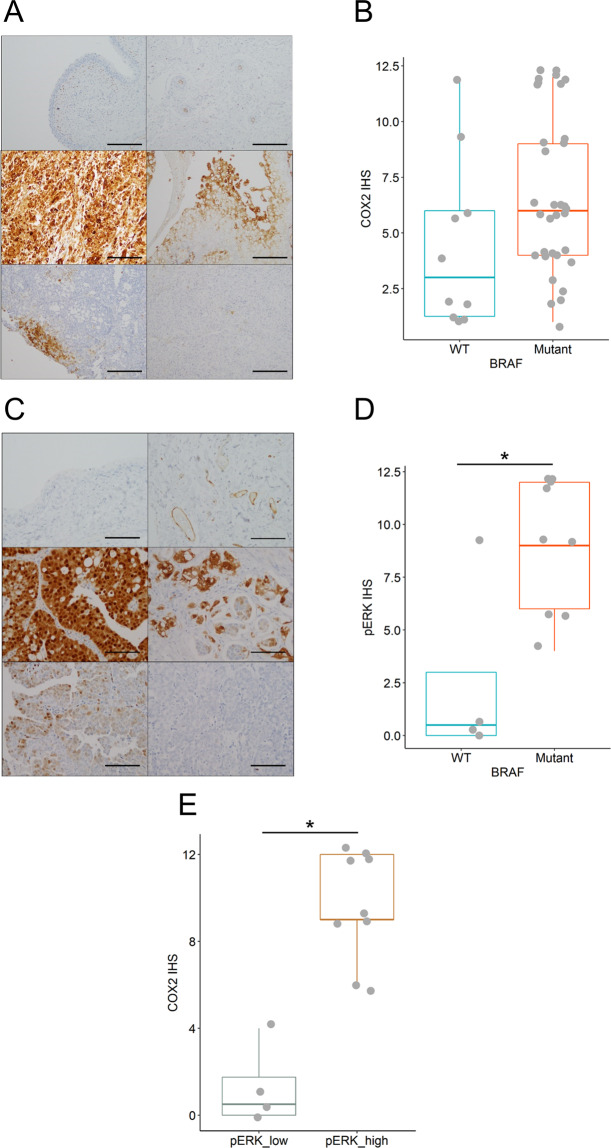
Levels of COX2 and pERK expression in cUC tissue. (**A**) Representative image of the COX2 expression in normal urothelium (top left) and blood vessel (top right) as negative and positive control, respectively, and cUC tissues (middle and bottom) at 100-fold magnification. Immunohistochemical score (IHS) was determined by a semi-quantitative method as described in Materials and Methods. IHSs of presented pictures were 12 (middle left), 9 (middle right), 6 (bottom left), 2 (bottom right), respectively. Scale bar: 200 μm. (**B**) A summarising box plot for COX2 expression in *BRAF* wild-type (WT; blue) and mutant cUC tissue (Mutant; red). Y axis represents IHS. p = 0.0569 (Wilcoxon rank sum test). (**C**) Representative image of the pERK expression in normal urothelium (top left) and blood vessel (top right) as negative and positive control, respectively, and cUC tissues (middle and bottom) at 200-fold magnification. IHSs of presented pictures were 12 (middle left), 9 (middle right), 4 (bottom left), 0 (bottom right), respectively. Scale bar: 100 μm. (**D**) A summarising box plot for COX2 expression in *BRAF* wild-type (WT; blue) and mutant cUC tissue (Mutant; red). Y axis represents IHS. *Indicates p < 0.05 (Wilcoxon rank sum test). (**E**) A summarising box plot for COX2 expression in cUC tissues which showed weak pERK expression with IHS < 6 (pERK_low; grey) and strong pERK expression with IHS ≥ 6 (pERK_high; orange). Y axis represents IHS for COX2. *Indicates p < 0.05 (Wilcoxon rank sum test).

To confirm our findings in patients, we performed immunohistochemical analysis of COX2 and pERK expression, and genotyping of the *BRAF* gene using the clinical tumour tissues from cUC patients. The levels of COX2 and pERK expression were analysed using a semi-quantitative method described in previous reports (Fig. 3 and Supplementary Fig. S4)^10,28–32^. Digital PCR-based *BRAF* genotyping identified *BRAF*^*V595E*^ mutation in 33 cUC tissues, while 10 tumours were *BRAF* wild-type. cUC tissues with *BRAF* mutation tended to have higher COX2 expression, although the difference was not statistically significant (p = 0.0569; Fig. 3A,B and Supplementary Fig. S4A). Moreover, phosphorylation of ERK was significantly stronger in *BRAF* mutant cUC tissue and strong phosphorylation of ERK was significantly associated with COX2 expression (Fig. 3C–E and Supplementary Fig. S4B).

### Involvement of p38 and JNK MAPK pathways in the regulation of COX2/PGE_2_

The results of the drug screening and the characterisation of the cUC cell lines also indicated that involvement of the other MAPK pathways in regulation of COX2/PGE_2_ in *BRAF*^*V595E*^ cUC cells. Therefore, additional *in vitro* experiments were performed using a p38 inhibitor (SB239063, SB) and a JNK inhibitor (SP600125, SP).

**Figure 4:**
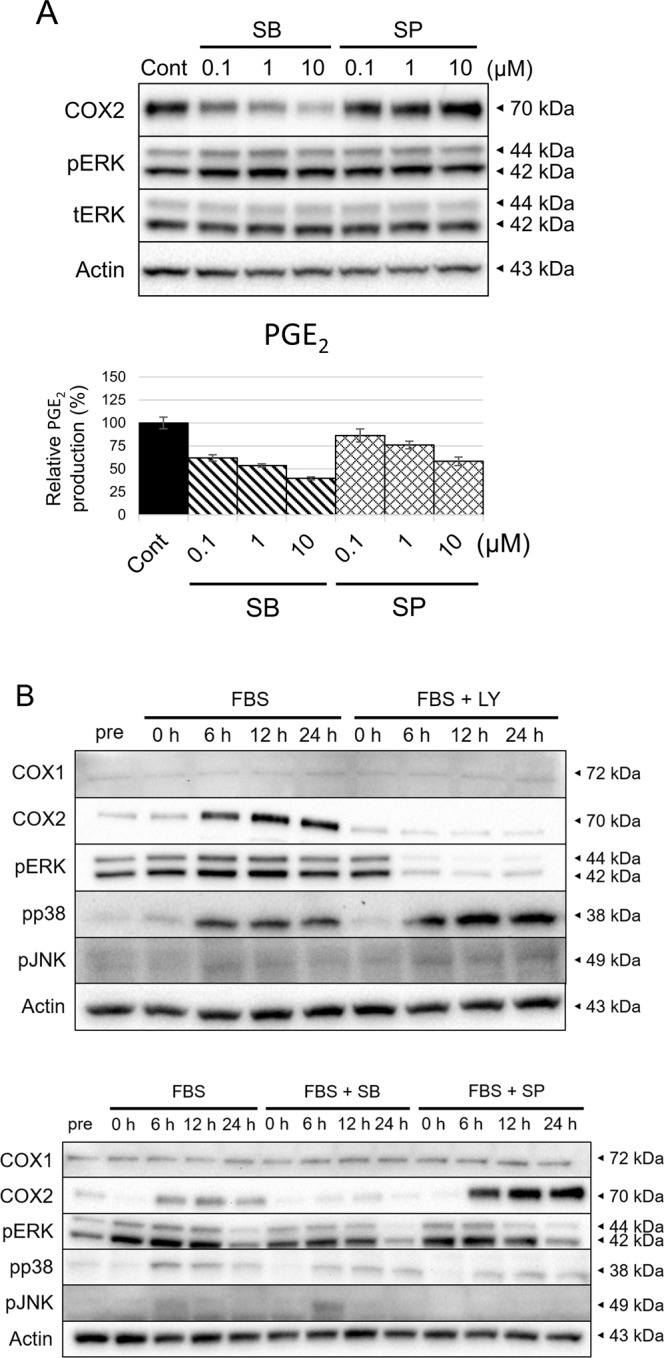
Contribution of p38 and JNK MAPK pathway to COX2 expression and PGE_2_ production in *BRAF*^*V595E*^ cUC. Protein levels in whole cell lysates were detected by Western blotting with Actin as loading control. (**A**) cUC cells (Sora) were treated with vehicle (Cont), SB239068(SB) and SP600125 (SP) for 12 h at indicated dose. Amount of PGE_2_ in culture medium was measured by enzyme-linked immunosorbent assay and normalised to cell number. Bar graph represents % control of PGE_2_ production. (**B**) cUC cells (Sora) were treated with foetal bovine serum (FBS) and LY3009120 (LY) at 1 μM, SB239068 (SB) at 10 μM or SP600125 (SP) at 10 μM after 24 h-starvation. Whole cell lysates were corrected before (pre) and after treatment for indicated time.

First, the *BRAF*^V595E^ Sora cell line was treated with the drugs at the varying concentrations for 12 h (Fig. 4A). As observed in the screening, inhibition of both pathways led to a reduction in PGE_2_ production in a dose-dependent manner. The two drugs showed the effects without changing the phosphorylation level of ERK, which indicated independency of the p38 and JNK pathways from the ERK pathway regarding the cellular control of PGE_2_. The involvement of p38 and JNK pathways were further generalised by the time-course experiment and demonstrations in the other cUC cell lines (Supplementary Fig. S5A and B). Inhibition of p38 and JNK was able to decrease PGE_2_ production in COX2-expressing *BRAF*^*V595E*^ cell lines without affecting ERK phosphorylation. Interestingly, throughout the experiments, inhibition of JNK increased COX2 at the protein level despite the decrease in PGE_2_ while p38 inhibition resulted in reduced COX2 expression. Inhibition of p38 and JNK pathways, as well as ERK pathway, did not change COX1 expression (Supplementary Fig. S6). These results suggested that the JNK pathway regulates PGE_2_ production in a COX1/COX2-independent manner while the ERK and p38 pathways were considered to exert the effects in a COX2-dependent manner.

To further explore the mechanism of action and relationship of the three MAPK pathways regarding control over cellular PGE_2_ production, starvation and refeeding experiments were performed in combination with inhibition of each pathway. As the p38 and JNK pathways are activated by external stimuli^33^, cells were refed with FBS after starvation to mimic them. Under starvation (“pre”), p38 and JNK were not activated while clear ERK phosphorylation was observed possibly because of the activating mutation in *BRAF* gene (Fig. 4B). It was also noted that the expression level of COX2 was low under starvation. Upon refeeding (“FBS”), COX2 expression was induced after 6 h and phosphorylation of p38 and JNK was also observed. When ERK MAPK pathway inhibitor was added with FBS (“FBS + LY”); ERK activation and COX2 induction were inhibited. No impact on COX1 and phosphorylated p38 and JNK was observed. Addition of p38 inhibitor at the same time with FBS (“FBS + SB”) also inhibited the induction of COX2 without affecting phosphorylation of ERK and JNK and expression of COX1. The ATP-competitive p38 inhibitor used in this study (SB239063) inhibited phosphorylation of downstream molecules by p38 without inhibiting the phosphorylation of p38 itself^34,35^. When refeeding was conducted with the JNK inhibitor (“FBS + SP”), phosphorylation of JNK was eradicated and the overexpression of COX2 was observed. There were no changes in COX1 expression and phosphorylation of ERK and p38.

This concludes that the three MAPK pathways independently regulate PGE_2_ production in the *BRAF* mutant cUC cells, not influencing each other. Moreover, ERK and p38 pathways mediate PGE_2_ production through COX2 expression. It seemed that activation of ERK MAPK itself does not induce COX2 overexpression, rather it maintains COX2 expression induced by other stimuli. Conversely, JNK may induce PGE_2_ production in COX1/2-independent manner.

## Discussion

The COX2/PGE_2_ axis plays a crucial role in tumour development. However, mechanisms of the aberrant expression of COX2/PGE_2_ in cUC with *BRAF*^*V595E*^ mutation remain to be elucidated. Influenced by our previous study which has found aberrant PGE_2_ production in *BRAF* mutant cUC cells, the series of *in vitro* and *in silico* analyses, as well as evaluation of the clinical samples from the patients, were performed to address this question. As a result, it was suggested that the activation of ERK MAPK pathway leads to upregulation of COX2/PGE_2_ in *BRAF*^*V595E*^ mutant cUC cells. In addition, the involvement of the ERK MAPK pathway in the regulation of COX2 expression was observed in the clinical samples of cUC tissues. Furthermore, it was suggested that the p38 and JNK pathways contribute to PGE_2_ production independently of the ERK pathway in *BRAF*^*V595E*^ cUC cells in a COX2-dependent and COX1/COX2-independent manner, respectively. ERK MAPK activation seems to maintain, rather than induce, COX2 expression induced by other stimuli. Further investigation thus is warranted for validation. Conclusively, our data revealed major mechanisms of PGE_2_ regulation in *BRAF*^*V595E*^ cUC cells. The results offer a new direction of anti-inflammatory strategy in clinical management of cUC as well as may explain the association relationship oncogenic mutation and tumour-associated inflammation.

**Figure 5:**
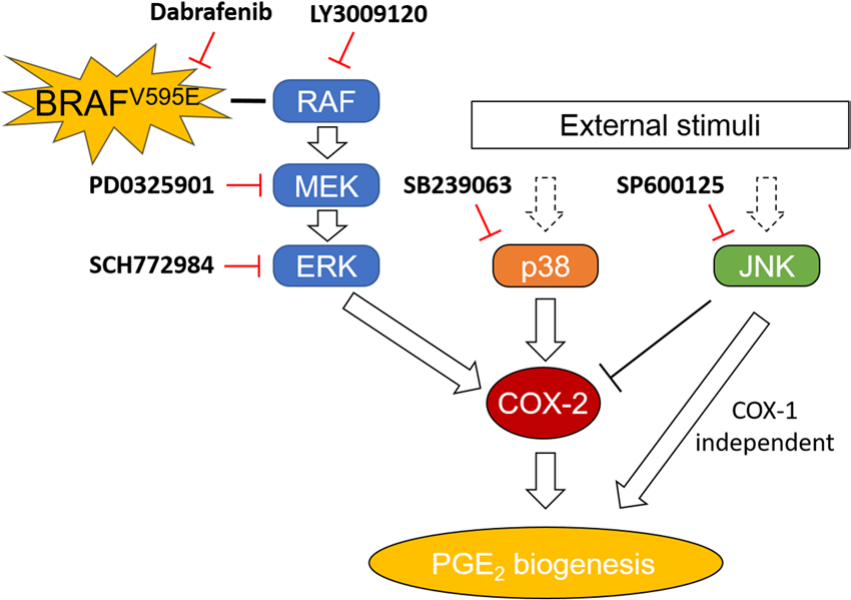
Schematic diagram of the involvement of ERK, p38 and JNK MAPK pathways in the regulation of COX2/PGE_2_ axis in *BRAF*^*V595E*^ mutant cUC.

Emerging evidences suggest that activation of ERK MAPK pathway in human cancer contributes to the regulation of COX2/PGE_2_ axis. A recent study suggested that COX2/PGE_2_ expression depends on the activation of the pathway in a *BRAF* mutant melanoma cell line established from a genetically engineered mouse model^36^. It has been suggested that ERK signalling activated by hepatocyte growth factor leads to increased COX2 expression in human colorectal cancer and non-small cell lung cancer^37,38^. In the present study, by pharmacological targeting of the multiple components in the pathway, it was shown that the ERK pathway plays a significant role in regulation of COX2 expression and PGE_2_ production in *BRAF* mutant cUC cells (Fig. 5). Expression of phospho-ERK was also correlated with COX2 expression in clinical samples. Simultaneously, the results of this study suggest that the ERK pathway is major, but not an only factor that regulates COX2/PGE_2_ axis in cUC. Although most cUC cell lines used in this study produced PGE_2,_ as a unique characteristic of cUC^13,39^, not all with activated ERK signalling showed overexpression of COX2. This observation suggested that there is an obvious heterogeneity among cUC cells in terms of regulation of COX2/PGE_2_ axis. Mechanistically, activation of ERK may not induce, rather maintain COX2 expression induced by other stimuli. As shown in Fig. 4B, under starvation, cells showed weak COX2 expression despite clear phosphorylation of ERK which possibly constitutively activated by mutated *BRAF*. When COX2 was strongly induced by refeeding with FBS, inhibition of the ERK MAPK pathway resulted in significant reduction of COX2/PGE_2_. Further, overexpression of COX2 and overproduction of PGE_2_ seemed to be caused by more than one pathway in cUC cells.

Possible candidates of such pathways were p38 and JNK pathways. p38 and JNK are members of the MAPK family and activated by environmental stress, including ultraviolet exposure, oxidative stress or inflammatory cytokines^33^. These pathways have been suggested to induce COX2 expression in inflammatory processes, such as rheumatoid arthritis or Parkinson’s disease, and in human cancer-promoting inflammation^40–42^. In this study, activation of the p38 pathway was involved in COX2 regulation and indicated to be essential to induce COX2 expression in the *BRAF*^*V595E*^ cUC cells based on the starvation and refeeding study. Addition of FBS induced phosphorylation of p38. Then, inhibition of p38 by the specific inhibitor decreased the COX2 level without affecting the COX1 expression and other MAPK pathways. Interestingly, induction of COX2 was almost completely inhibited by either of p38 or ERK MAPK inhibition. ERK MAPK inhibition did not interfere with p38 MAPK pathway. In addition, activation of ERK MAPK did not induce COX2 expression as discussed above (“pre” state when cells are under starvation). One hypothesis for this observation is a cooperative regulation of COX2 by the two pathways. Souza *et al*. reported that the ERK MAPK pathway stabilises COX2 mRNA induced by the p38 MAPK pathway in human Barrett’s-associated adenocarcinoma treated with the low pH environment^43^. In our cell line characterization, overproduction of PGE_2_ were found mostly in the cell lines with both p38 and ERK activation (TCCUB, Sora, Nene, and NMTCC). This hypothesis needs to be further examined in cUC by simultaneous inhibition of both pathways with pharmacological or, more preferentially, genetic approaches. The importance of the p38 pathway in regulation of COX2 also needs to be validated in clinical samples.

Conversely, JNK inhibition upregulated COX2 at the protein level despite a significant decrease in PGE_2_ production. Refeeding with FBS induced phosphorylation of JNK, which was counteracted by JNK inhibitor. The drug did not have clear impact on either of ERK, p38 or COX1. These results indicated that JNK promotes PGE_2_ production in the cUC cells, independent of ERK and p38, which involves regulation of neither of COX1 and COX2. A previous report indicated that the JNK pathway can regulate PGE_2_ production in a COX-independent manner. In the paper, JNK pathway activated by TNF-α was involved in the gene expression of one of the PGE_2_ synthases, mPGES-1, in gingival fibroblasts^44^. The exact mechanism of action of JNK pathway in cellular PGE_2_ production can be further explored by a combination of a specific JNK activator^33^ and an unbiased comprehensive approach such as transcriptome analysis and drug screening. Simultaneous inhibition of three MAPK pathways using specific RNA interference will also clarify the association between the pathways.

The heterogeneity observed in cUC cell lines and clinical samples could be explained by two hypotheses. One is that complexed regulation of PGE_2_ production by the three different MAPK pathways. It seemed that activation of one of them is not sufficient to induce and maintain the eicosanoid production at an increased level. Another hypothesis is that there can be multiple pathways to trigger activation of ERK MAPK pathway. In cUC clinical samples, the association between *BRAF* genotype and COX2 expression was not clear, partly because some wild-type tumours showed increased COX2 expression. There is a similar observation in human medicine. Although COX2 plays a critical role in *BRAF* mutant human colorectal cancers, expression of COX2 is not associated with the presence of *BRAF* mutation^45^. This may be because there is another major driver mutation of KRAS in human CRC, which also leads to constitutive activation of ERK MAPK pathway. In cUC, overexpression of HER2 has been recently reported and this can contribute to activation of the ERK MAPK pathway^46,47^. Therefore, a future comprehensive study will need to assess *BRAF* genotype, HER2 expression, activation of the MAPK pathways and COX1/2 expression simultaneously. From this study, since *BRAF* mutation was associated with pERK IHS score and pERK IHS score was clearly associated with COX2 expression, we believe that *BRAF* mutation would contribute to the overexpression of COX2 in cUC although the direct causal relationship of them should be further examined. In a recent study, the association between *BRAF* mutation and COX2 expression was suggested in cUC tissues in specific dog breeds^48^.

One major hypothesis built primarily on *in silico* analysis, was that *BRAF*^*V595E*^ mutation directly causes COX2 overexpression via upregulation of the ERK/MAPK pathway. In this study, we focused on the importance of ERK and other MAPK pathways in regulation of COX2/PGE_2_ in *BRAF* mutant cUC. The direct causal association between the mutation and COX2 expression should be further validated by gain-of-function and loss-of-function studies using canine cells. Activation in *BRAF* gene is known to induce aberrant activation of ERK MAPK pathways in mice, human and canine cells^14,16,19,36^. Moreover, in several human tumours, it has been suggested that the COX2/PGE_2_ axis is affected by oncogenic mutations including *BRAF*^*V600E*^ ^49,50^. Considering the important role of the COX2/PGE_2_ axis in the pro-tumoural microenvironment and the unique vulnerability of cUC to anti-COX2 therapies, *BRAF*^*V595E*^ cUC would serve as a valuable model to explore the possible association between oncogenic mutation and pro-tumoural microenvironment.

cUC is a unique tumour often managed with NSAID monotherapy, indicating the importance of the COX2/PGE_2_ axis in the development and progression of cUC^9^. Since no direct cytotoxicity was observed in our previous study^13^, the therapeutic effect of NSAIDs was considered to be indirect, possibly on tumour microenvironment. This hypothesis was further supported in this study by the fact that no correlation was observed between suppression of PGE_2_ production and cell growth. Although a causal relationship between *BRAF* mutation and PGE_2_ production is further to be determined, the findings of this study may suggest that driver mutation can render tumour cells malignant phenotypes through modulation of the microenvironment to a pro-tumoural niche rather than direct growth advantages.

In conclusion, our study revealed that activation of the ERK MAPK pathway mediated overexpression of COX2 and PGE_2_ production in *BRAF*^*V595E*^ cUC cells. In addition, COX2 expression was cooperatively regulated by the p38 MAPK pathway. Oppositely, the JNK pathway was suggested to facilitate PGE_2_ production in a COX1- /COX2-independent manner (Fig. 5). By understanding how significantly and differently each MAPK pathway regulates PGE_2_ production in cUC, these findings may open up the possibility of PGE_2_-targeted therapy replacing the current standard treatment with NSAIDs, or COX inhibitors. The major hypothesis to be answered in future studies is direct causal relationship between the *BRAF* mutation and COX2 expression. This may help us to understand heterogeneity in COX2 expression in cUC and how tumour cells establish pro-tumoural environment surrounding them. As cUC has its uniqueness of the clinical vulnerability to the COX inhibitors, we believe that *BRAF*^*V595E*^ cUC serve as a good model to study relationship between *BRAF* mutation and COX2/PGE_2_ axis, or driver mutation and pro-tumoural microenvironment, from a wider perspective.

## Methods

### Cell culture

Eight cUC cell lines, TCCUB, Sora, Love, Nene (originally established in our laboratory), NMTCC, LTCC, MCTCC and OMTCC (kindly provided by Hokkaido University) were used in this study^13,51,52^. Each cell line was maintained in RPMI-1640 supplemented with 10% heat-inactivated foetal bovine serum (FBS) and 5 mg/L gentamicin at 37 °C in a humidified atmosphere with 5% CO_2_.

### Drugs

For *in vitro* drug screening, the SCADS inhibitor kits (kit 1, ver 3.3; kit 2, ver 2.0; kit 3, ver 1.6; kit 4, ver 2.3) were kindly provided by Molecular Profiling Committee, Grant-in-Aid for Scientific Research on Innovative Areas “Platform of Advanced Animal Model Support” from The Ministry of Education, Culture, Sports, Science and Technology, Japan (KAKENHI 16H06276). For the following experiment, dabrafenib (BRAF inhibitor), PD0325901 (MEK inhibitor) and SCH772984 (ERK inhibitor) were purchased from Selleck. LY3009120 (pan-RAF inhibitor) was purchased from Cayman Chemical. SB239063 (p38 inhibitor) and SP600125 (JNK inhibitor) were purchased from Sigma-Aldrich. These inhibitors were reconstituted in DMSO and stored at −20 °C or −80 °C.

### PGE_2_ measurement

PGE_2_ concentration in culture supernatant was measured using enzyme-linked immunosorbent assay kit (Cayman Chemical) according to the manufacturer’s instructions. To normalise the amount of PGE_2_ to the cell density, direct cell count or sulforhodamine B (SRB) assay was performed^53^.

### *In vitro* drug screening

Sora cells were seeded in a 96-well plate at a density of 11,000 cells per well. After 24-h incubation, the culture medium in each well was discarded, and fresh medium containing the drugs from the SCADS inhibitor kits was added at a final concentration of 10 µM. Following a 12-h incubation, the culture supernatants were collected. After normalising PGE_2_ production to cell density as mentioned above, percentage change in PGE_2_ production from control was calculated.

### Growth inhibition

To compare with the changes in PGE_2_ production, data regarding cell growth inhibition after treatment with the inhibitors from the SCADS inhibitor kits were obtained from our previous study, where the detailed protocol is described^52^. Briefly, Sora cells were treated with the drugs from the SCADS inhibitor kits at 10 µM as described in *In vitro drug screening* section. After 72-h incubation, cell densities were determined by SRB assay.

### Protein detection

cUC cells were seeded in serum-free medium and incubated for 24 h as in our previous report (Supplementary Table S3)^52^. After 24-h serum starvation, cUC cells were treated with final concentration of 10% FBS and ERK MAPK, p38 MAPK or JNK MAPK inhibitors as indicated in each figure. Following treatment for the indicated times, cells were lysed for 30 min on ice in RIPA buffer consisting of 50 mM Tris-HCl, 150 mM NaCl, 5 mM EDTA, 0.1% sodium dodecyl sulphate, 1% Triton-X, 10 mM NaF, 2 mM NaVO_4_ and complete protease inhibitor cocktail (Roche Diagnostics). After centrifugation at 12000 g, 4 °C for 20 min, protein concentrations were measured using bicinchoninic acid protein assay kit. Equal amounts (10 µg) of total protein were separated by 10% sodium dodecyl sulphate-polyacrylamide gel electrophoresis and transferred to polyvinylidene difluoride membranes. The membranes were blocked with 5% skim milk in Tris-buffered saline containing 0.1% Tween (TBST) for 1 h at room temperature to avoid non-specific antibody binding. Further, the membranes were incubated overnight at 4 °C with each primary antibody (Supplementary Table S4). The membranes were then washed with TBST and incubated with horseradish peroxidase-conjugated anti-mouse or anti-rabbit secondary antibody (1:10,000) from GE Healthcare for 1 h at room temperature. Protein signal was developed with a chemiluminescent system (Merck Millipore) and captured with an imaging system (BioRad Laboratories).

### *In silico* BRAF gain-of-function analysis

Data search was performed using two public database (PubMed and GREIN) maintained by National Institute of Health, the United States^23^. The databases were accessed on 1/27/2020 and queries were performed with the term of “BRAF” and “transfect”. Four studies were identified with publicly available data (Supplementary Table S2)^24–27^. For the study by Becker *et al*., the fold change data was collected from the table in the original manuscript^24^. For the other studies, the normalised expression values were retrieved through GREIN. The expression values of the genes in the transfected cells were further normalised by the expression values in the parental cells and presented as the fold changes.

### Immunohistochemistry

Formalin-fixed paraffin-embedded (FFPE) cUC tissues were retrospectively evaluated. Surgically resected cUC tissue samples (n = 43) were obtained from the archival collection of the Veterinary Medical Centre of the University of Tokyo (samples collected from 2009 to 2012 and 2015 to 2016) and Veterinary Medical Teaching Hospital of Nippon Veterinary and Life Science University (from 2003 to 2017). The clients from respective hospitals provided informed consent for the use of these samples for this study. cUC tissues of 4 μm thickness were deparaffinised in xylene and hydrated in graded alcohol. Antigen retrieval was performed for 10 min at 121 °C in citrate buffer (pH 6.0). The sections were then treated with 3% hydrogen peroxide for 30 min at room temperature. After blocking with 5% normal goat serum in TBST for 1 h at room temperature, the sections were incubated overnight with 1:100 of anti-COX2 mouse monoclonal antibody (BD Bioscience) or 1:400 of anti-phospho-ERK1/2 (Thr202/Tyr204) rabbit monoclonal antibody (Cell Signaling Technology) at 4 °C in a humidified chamber. Subsequently, the sections were rinsed with TBST and then incubated with EnVision polymer reagent for mouse (Dako) for 60 min at room temperature. After rinsing with TBST again, peroxidase reactions were developed for 3 min with 3,3′-diaminobenzidine (Dako). The sections were counterstained with haematoxylin.

### BRAF genotyping

*BRAF* genotype of cUC tissue was determined as described in a previous study^52^. Briefly, gDNA was extracted from FFPE sections of cUC tissue using Qiaamp DNA FFPE Tissue Kit (Qiagen). *BRAF*^*V595E*^ and wild-type *BRAF* gene were amplified with Taqman probe and signal detection was performed using QuantStudio 3D Digital PCR System (Thermo Fisher Scientific).

### Immunohistochemical evaluation

For scoring COX2 and phospho-ERK (pERK) expression in cUC tissue, a semi-quantitative immunohistochemical score (IHS) system described in a previous report was used^10,28–32^. Briefly, the percentage of COX2- or pERK-positive tumour cells in the entire section and their signal intensity were evaluated when viewed at 100x and 200x magnification, respectively. Positivity was graded as 1 = <1%, 2 = 1%–9%, 3 = 10%–50%, 4 = >50% for COX2 and as 0 = <5%, 1 = 5%–25%, 2 = 26%–50%, 3 = 51%–75%, 4 = >75% for pERK, and intensity was graded as 0 = no staining, 1 = mild staining, 2 = moderate staining, 3 = strong staining for COX2 and pERK. IHS was obtained by multiplying positivity score and intensity score.

### Statistical analysis

Statistical analysis was performed using R software (https://www.R-project.org/). To determine enriched pathways revealed in drug screening, all of the 331 compounds were divided into 98 groups according to their targeting pathways, and p value for each pathway was calculated using hypergeometric distribution. Multiple comparison was adjusted by Benjamini–Hochberg method and the FDR cut-off was set as 0.1. To test the effect of ERK MAPK inhibition on PGE_2_ production, Dunnett’s multiple comparison test was used. Difference in the IHS score depending on the *BRAF* genotype or pERK IHS score (pERK_high; IHS ≥ 6 vs pERK_low; IHS < 6) was calculated using Wilcoxon rank sum test. All values are shown as the mean value ± standard deviation. p values <0.05 were considered statistically significant.

## Supplementary information

Supplementary information

Supplementary information2

## Data Availability

The data obtained during the current study will be available from the corresponding author upon reasonable request.
