# Author Correction: Aberrant expression of the COX2/PGE_2_ axis is induced by activation of the RAF/MEK/ERK pathway in BRAF^V595E^ canine urothelial carcinoma

**DOI:** 10.1038/s41598-020-75949-y

**Published:** 2020-10-28

**Authors:** Ryohei Yoshitake, Kohei Saeki, Shotaro Eto, Masahiro Shinada, Rei Nakano, Hiroshi Sugiya, Yoshifumi Endo, Naoki Fujita, Ryohei Nishimura, Takayuki Nakagawa

**Affiliations:** 1 Laboratory of Veterinary Surgery, Graduate School of Agricultural and Life Sciences, University of Tokyo, 1-1-1, Yayoi, Bunkyo-ku, Tokyo, 113-8657 Japan; 2 Laboratory of Veterinary Biochemistry, Department of Veterinary Medicine, Nihon University College of Bioresource Sciences, 1866 Kameino, Fujisawa, Kanagawa 252-0880 Japan

Correction to: *Scientific Reports* 10.1038/s41598-020-64832-5, published online 8 May 2020

The original version of the Article contained errors in the spelling of the authors Ryohei Yoshitake, Kohei Saeki, Shotaro Eto, Masahiro Shinada, Rei Nakano, Hiroshi Sugiya, Yoshifumi Endo, Naoki Fujita, Ryohei Nishimura and Takayuki Nakagawa.

These errors have now been corrected in the PDF and HTML versions of the Article and in the accompanying Supplementary Information file.

