# Supplementary material for: Aberrant expression of the COX2/PGE2 axis is induced by activation of the RAF/MEK/ERK pathway in BRAFV595E canine urothelial carcinoma
Source: Sci Rep. 2020 May 8;10:7826. doi: 10.1038/s41598-020-64832-5 (PMC7210937; doi:10.1038/s41598-020-64832-5)
Supplement: Supplementary file 1 — Supplementary information [file 41598_2020_64832_MOESM1_ESM.pdf]

Aberrant expression of the COX2/PGE_2 axis is induced by activation of the RAF/MEK/ERK pathway in $BRAF^{V595E}$ canine urothelial carcinoma

Ryohei Yoshitake [a], Kohei Saeki [a, *], Shotaro Eto [a], Masahiro Shinada [a], Rei Nakano [b], Hiroshi Sugiya [b], Yoshifumi Endo [a], Naoki Fujita [a], Ryohei Nishimura [a], Takayuki Nakagawa [a]

[a] *Laboratory of Veterinary Surgery, Graduate School of Agricultural and Life Sciences, University of Tokyo, 1-1-1, Yayoi, Bunkyo-ku, Tokyo 113-8657, Japan*
[b] *Laboratory of Veterinary Biochemistry, Department of Veterinary Medicine, Nihon University College of Bioresource Sciences, 1866 Kameino, Fujisawa, Kanagawa, 252-0880, Japan*

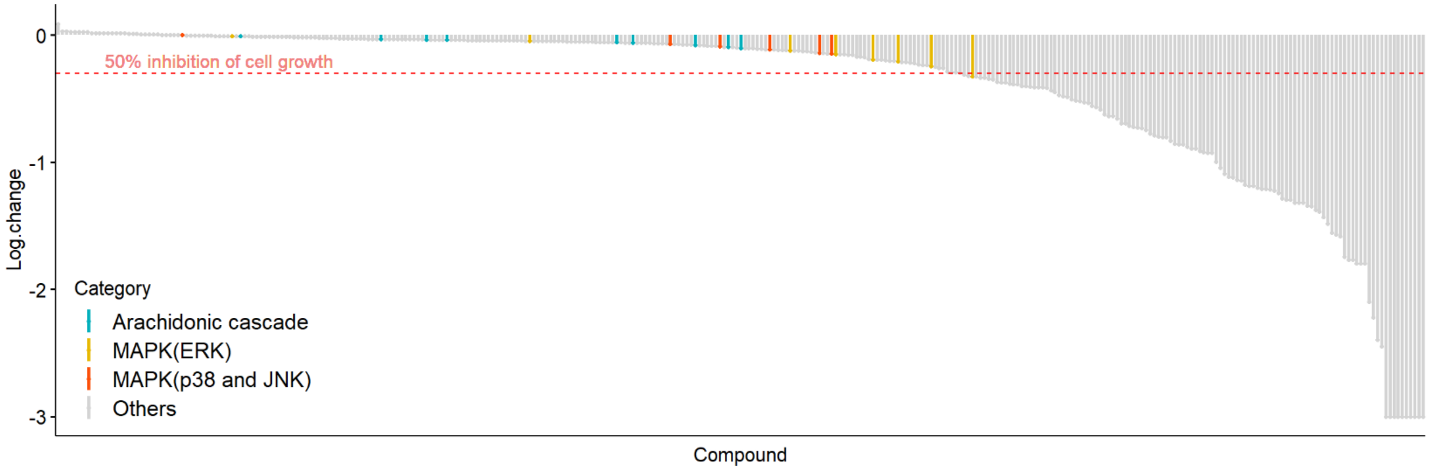

Supplementary Figure S1. Cell growth in cUC cells (Sora) during drug screening. Y axis represents log 10 values of changes in cell density for each inhibitor (n = 331). The drugs which belong to selected pathways are coloured as indicated.

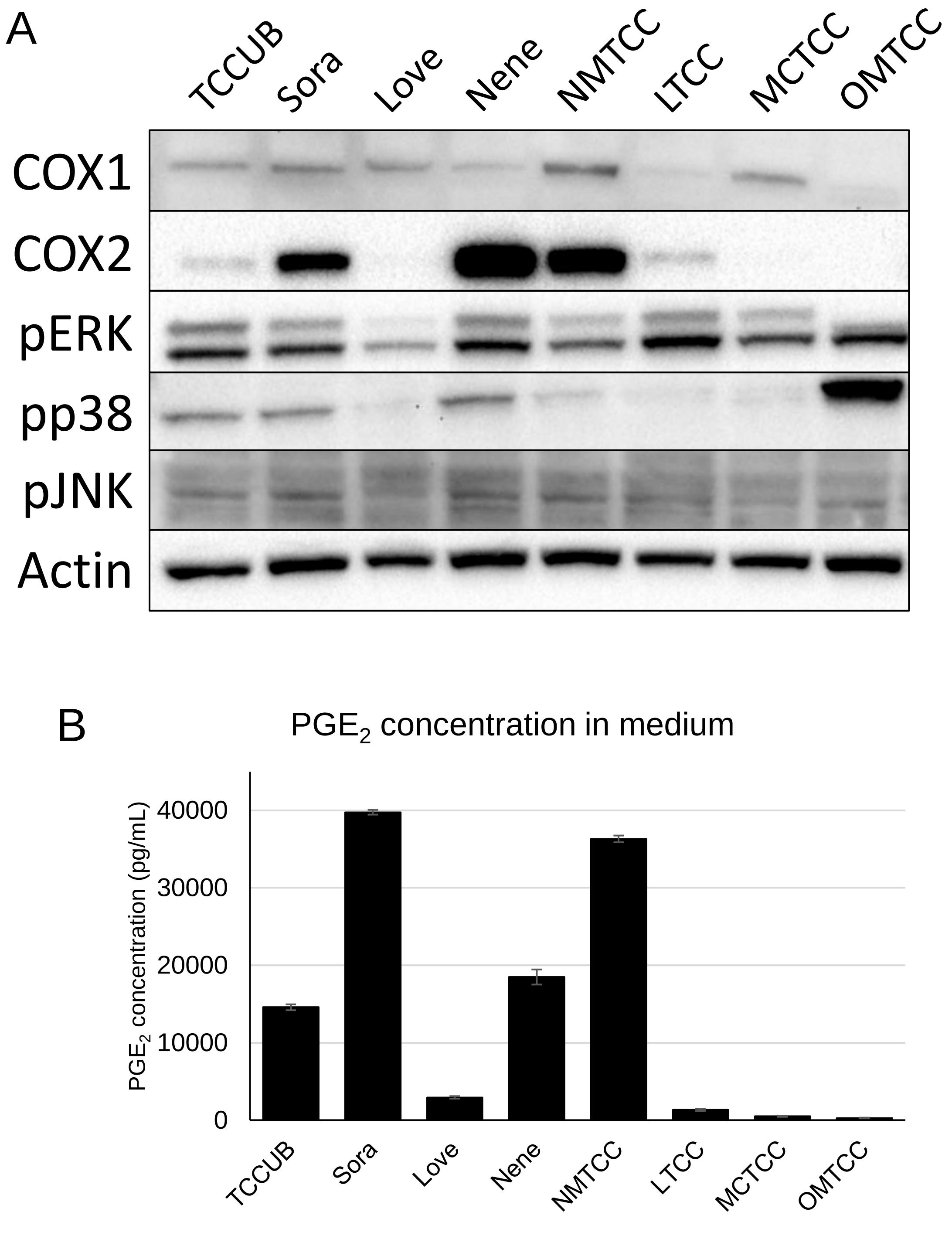

Supplementary Figure S2. Characteristics of the cUC cell lines used in this study. (A) Basal expression of the proteins associated with PG production and MAPK pathways. (B) Basal PGE_2 production. cUC cell lines (TCCUB, Sora, Love, Nene, NMTCC, LTCC, MCTCC and OMTCC) were seeded in serum free medium and incubated for 24 h. After 24 h serum starvation, cUC cells were treated with final concentration of 10% foetal bovine serum and incubated for further 24 h. Protein levels in whole cell lysate were detected by Western blotting with Actin as loading control. PGE_2 concentration in culture supernatant was measured using enzyme-linked immunosorbent assay. Data are presented as mean ± SD of three experiments.

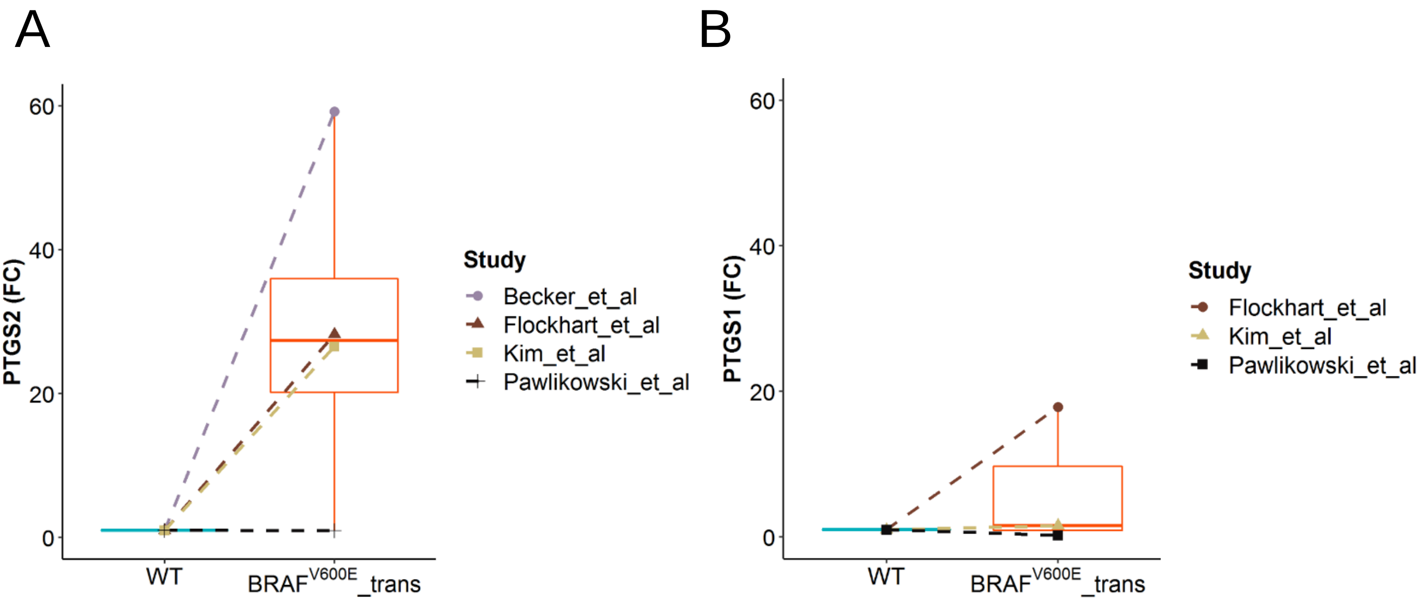

Supplementary Figure S3. Change in (A) *PTGS2* and (B) *PTGS1* expression by mutant *BRAF* transfection. Data was obtained from four studies in public databases (PubMed and GREIN). Y axis represents the fold change (FC) of the gene expression value of the *BRAF*^{V600E} transfected cells (BRAF^{V600E}_trans; red) normalised by that of the parental cells (WT; blue).

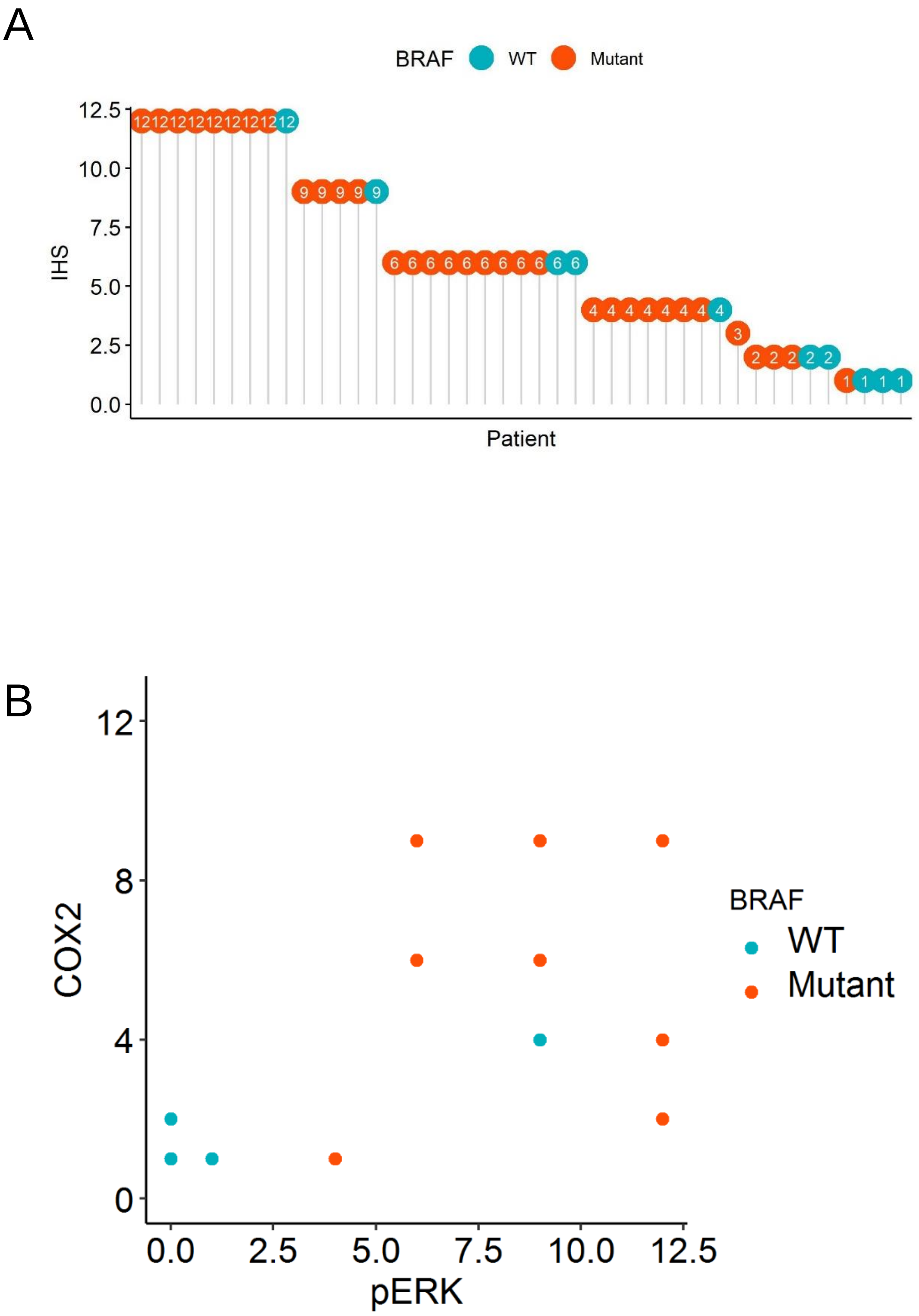

Supplementary Figure S4. (A) Breakdown of *BRAF* genotype and immnohistochemical score (IHS) for COX2 in individual patients. Y axis represents IHS in each patient with wild-type *BRAF* (WT; blue) and mutant *BRAF*^{V595E} (Mutant; red). (B) Relationship of COX2 IHS with *BRAF* genotype and pERK. X axis and Y axis represent the IHS in each patient for pERK and COX2, respectively. Colour represents the BRAF genotype as described above. IHS for COX2 and pERK was determined by a semi-quantitative method as described in Materials and Methods.

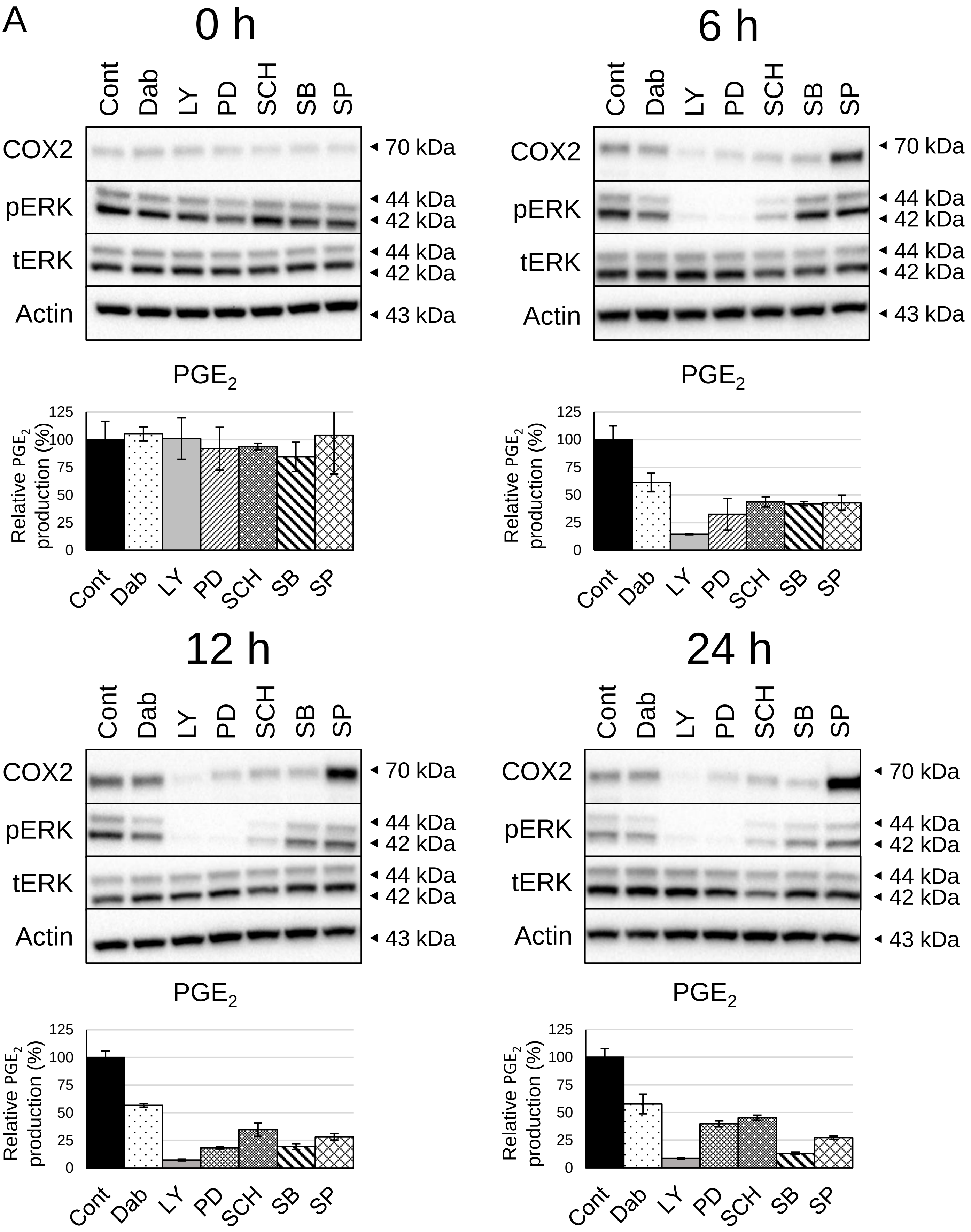
A
0 h
6 h
12 h
24 h
Cont
Dab
LY
PD
SCH
SB
SP
COX2
pERK
tERK
Actin
70 kDa
44 kDa
42 kDa
43 kDa
PGE2
Relative PGE2 production (%)
125
100
75
50
25
0

B

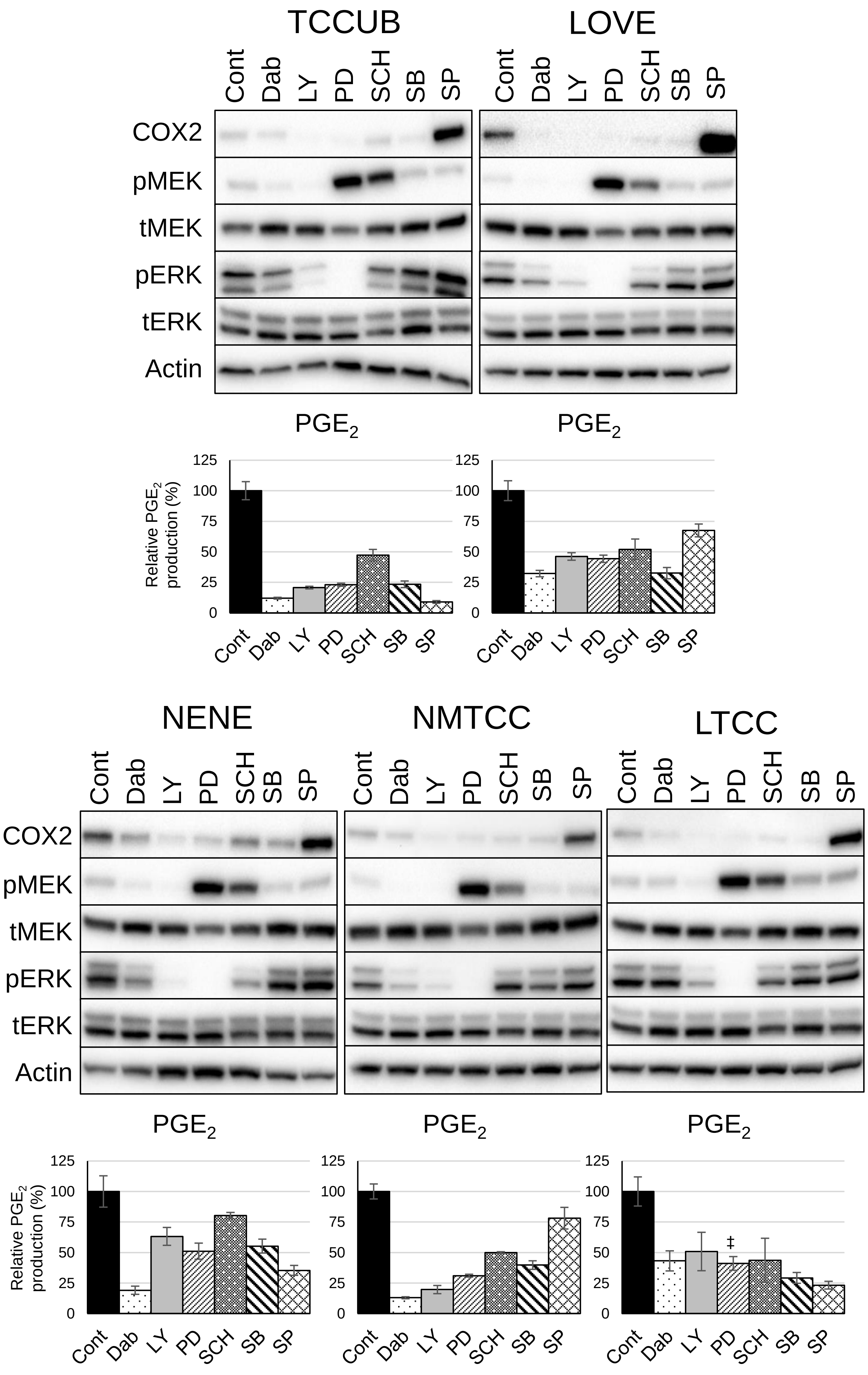

TCCUB
LOVE
Cont
Dab
LY
PD
SCH
SB
SP
COX2
pMEK
tMEK
pERK
tERK
Actin
PGE2
Relative PGE2 production (%)
125
100
75
50
25
0
NENE
NMTCC
LTCC
‡

B (cont'd)

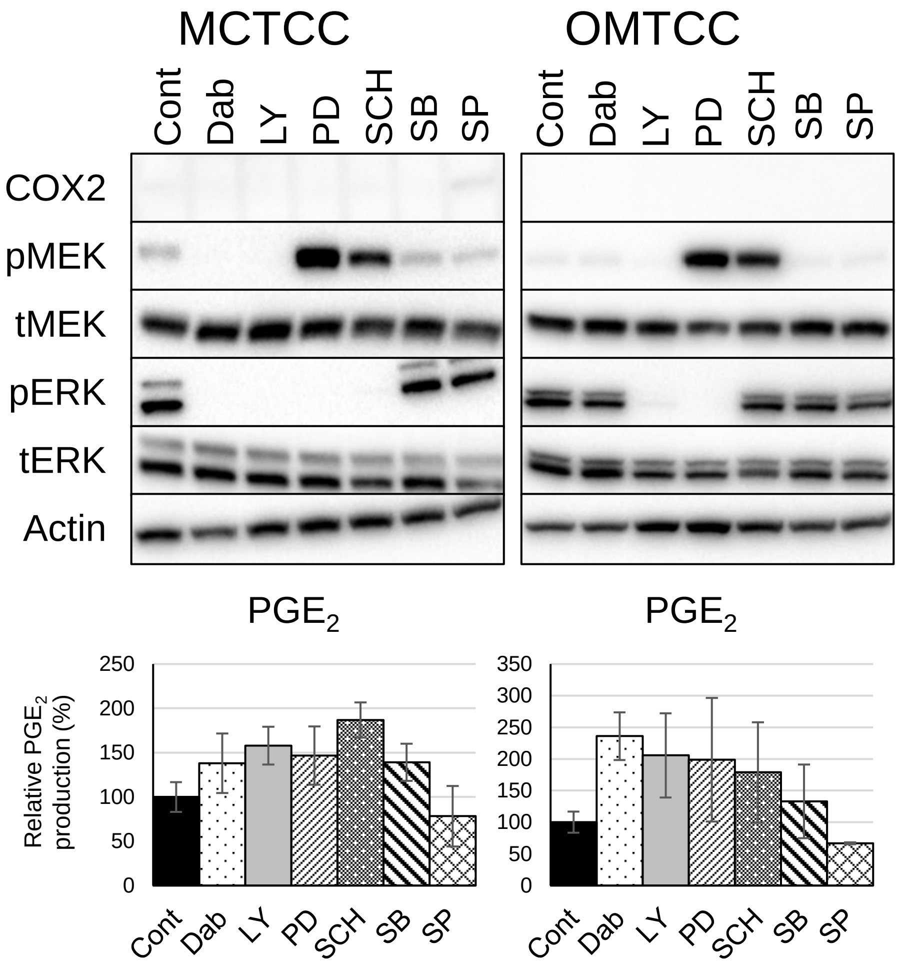

Supplementary Fig. S5. Effect of p38 and JNK pathway inhibition on COX2 expression and PGE_2 production. Protein levels in whole cell lysate were detected by Western blotting with Actin as loading control. Amount of PGE_2 in culture medium were measured by enzyme linked immunosorbent assay and corrected to cell number. Bar graph represents % control of PGE_2 production. (A) Whole image of the membrane and PGE_2 production shown in Figure. 2A. cUC cells (Sora) were treated with vehicle (dimethyl sulfoxide; Cont) and inhibitors of BRAF (Dabrafenib; Dab), pan-RAF (LY3009120; LY), MEK (PD0325901; PD), ERK (SCH772984; SCH), p38 (SB239063; SB) and JNK (SP600125; SP) at 1 μM for indicated time. (B) Whole image of the membrane and PGE_2 production shown in Figure. 2C. The seven cUC cell lines (TCCUB, Love, Nene, NMTCC, LTCC, MCTCC and OMTCC) were treated with vehicle (Cont), Dabrafenib (Dab) at 10 μM, LY3009120 (LY) at 1 μM, PD0325901 (PD) at 10 μM, SCH772984 (SCH) at 10 μM, SB239068(SB) at 10 μM and SP600125 (SP) at 10 μM for 12 h. Data are presented as mean ± SD of three experiments.

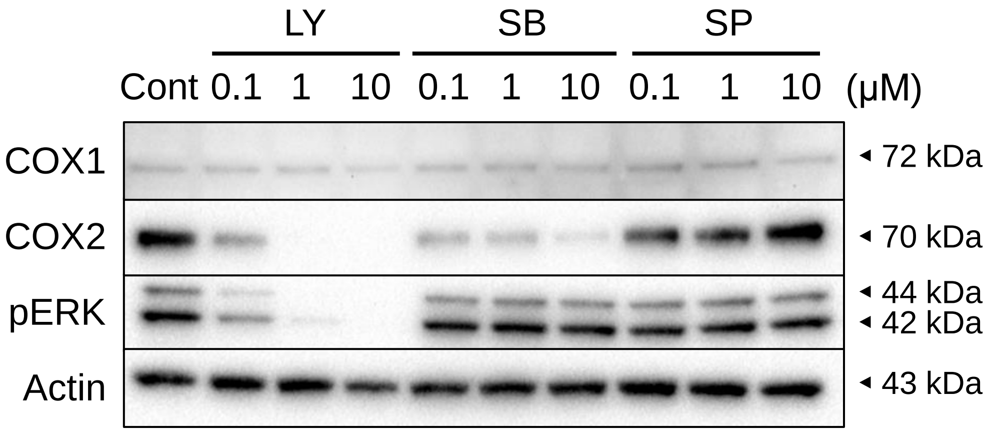

Supplementary Figure S6. Effect of ERK, p38 and JNK MAPK inhibition on COX1 and COX2 expression. Protein levels in whole cell lysates were detected by Western blotting with Actin as loading control. cUC cells (Sora) were treated with vehicle (dimethyl sulfoxide; Cont) and inhibitors of BRAF (Dabrafenib; Dab), pan-RAF (LY3009120; LY), MEK (PD0325901; PD) and ERK (SCH772984; SCH) for 12 h at indicated dose.

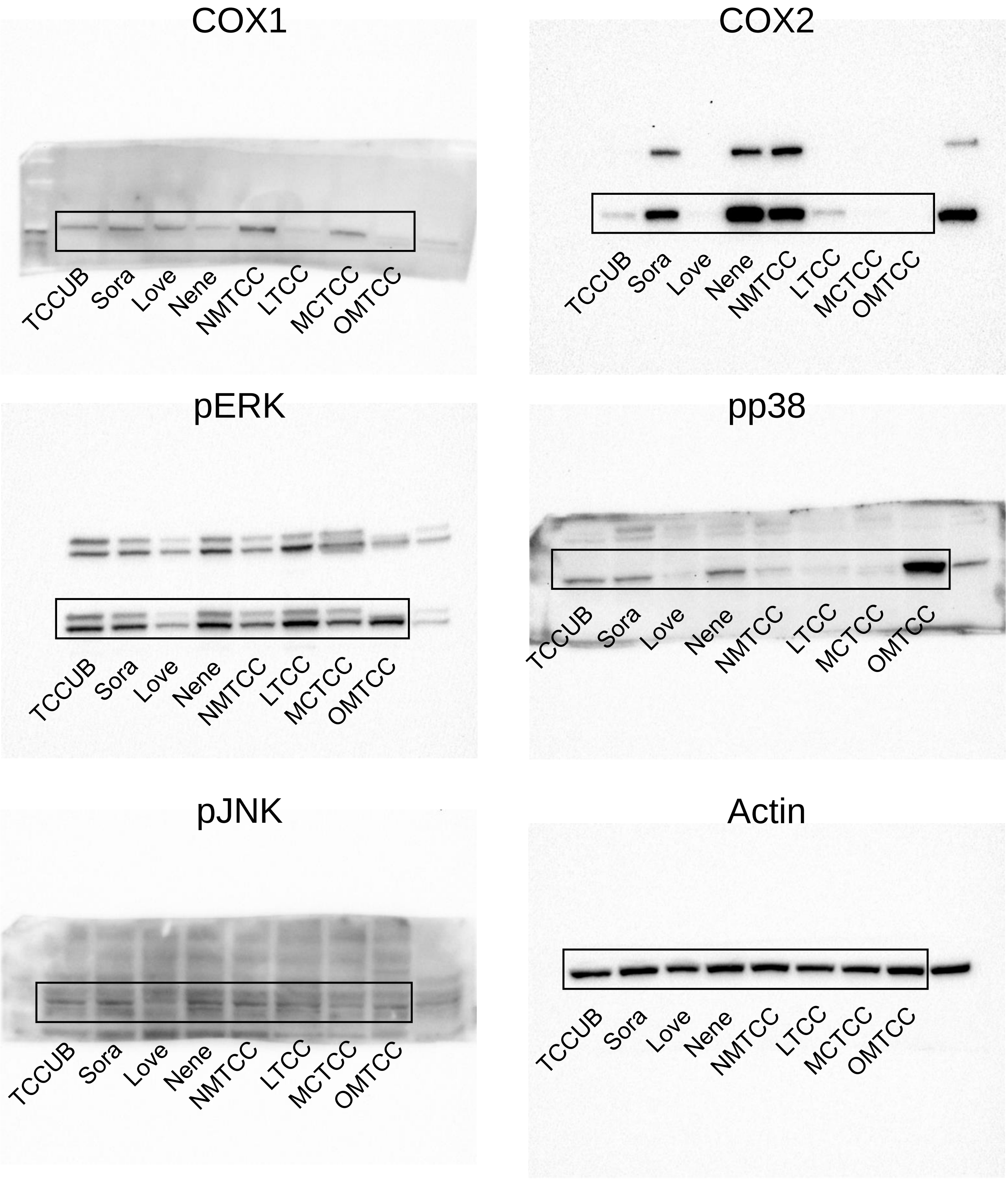

Supplementary Figure S7. Uncropped image of Western blots for Supplementary Figure S2A. cUC cell lines (Sora, TCCUB, Love, Nene, LTCC, MCTCC and OMTCC) were seeded in serum free medium and incubated for 24 h. After 24 h serum starvation, cUC cells were treated with final concentration of 10% foetal bovine serum and incubated for further 24 h.

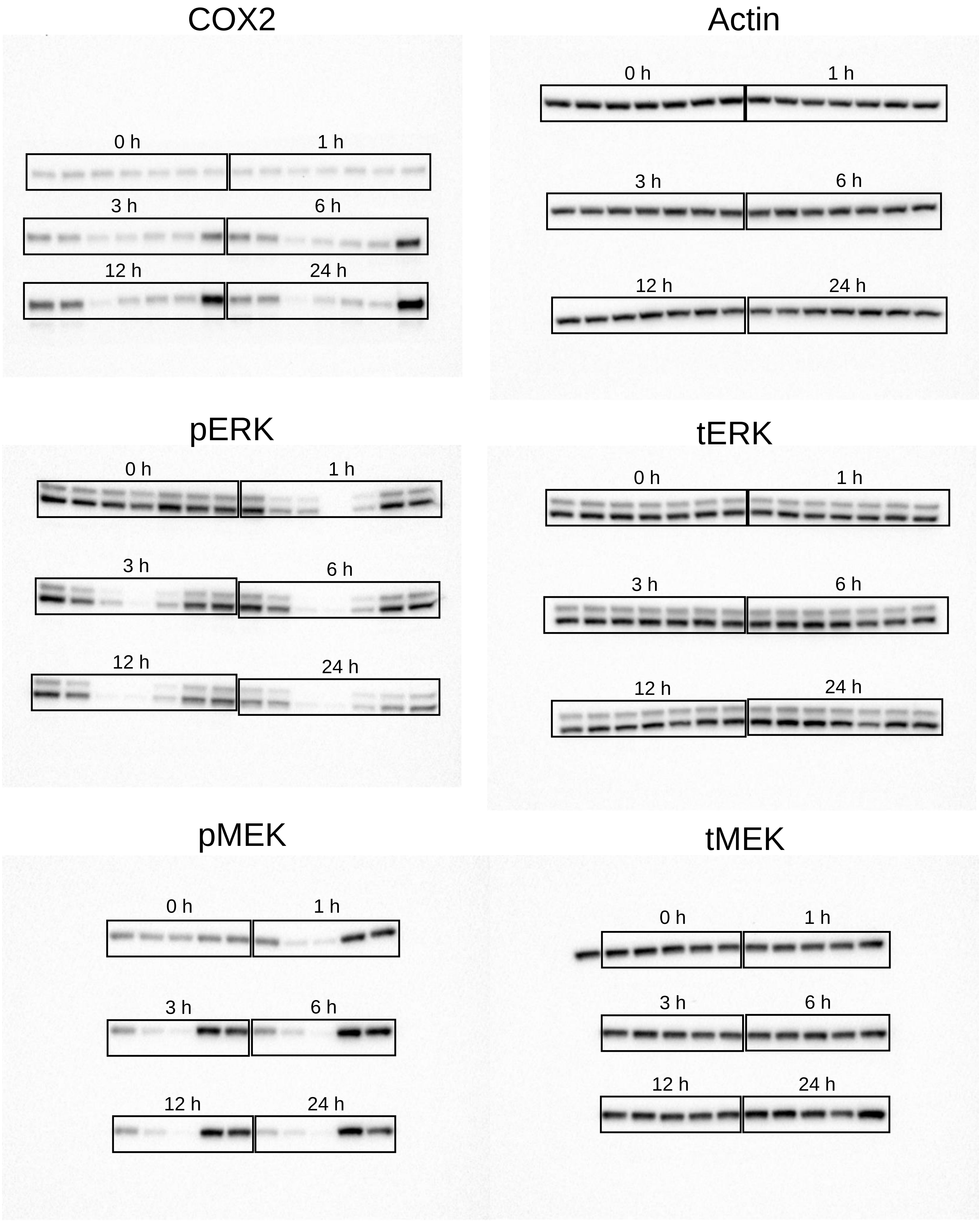

Supplementary Figure S8. Uncropped image of Western blots for Figure 2A and Supplementary Figure S5A. cUC cells (Sora) was treated with vehicle (dimethyl sulfoxide) and inhibitors of BRAF (Dabrafenib), pan-RAF (LY3009120), MEK (PD0325901), ERK (SCH772984), p38 (SB239063) and JNK (SP600125) at 1 µM for indicated time.

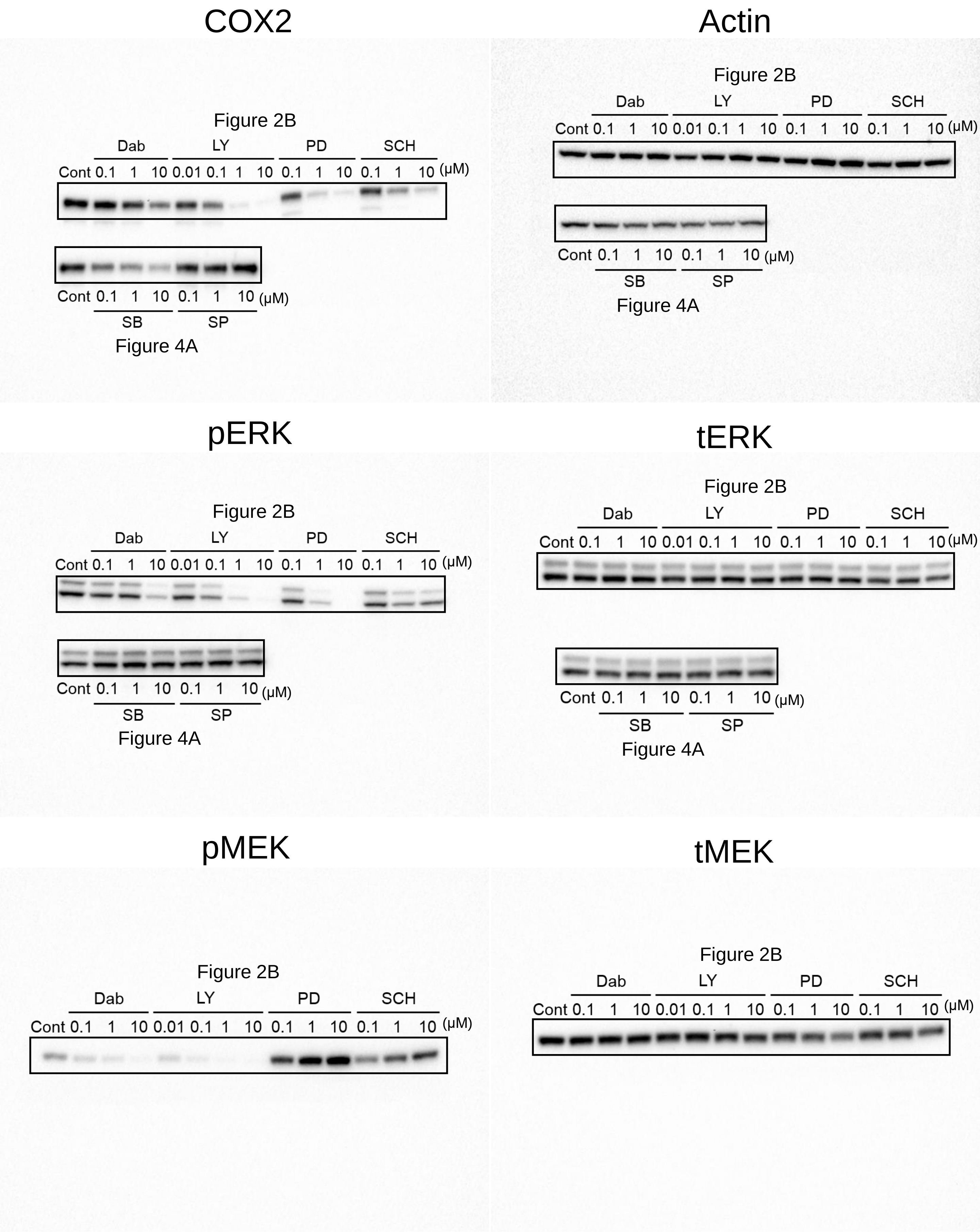

Supplementary Figure S9. Uncropped image of Western blots for Figure 2B and 4A. cUC cells (Sora) was treated with vehicle (dimethyl sulfoxide; Cont) and inhibitors of BRAF (Dabrafenib; Dab), pan-RAF (LY3009120; LY), MEK (PD0325901; PD), ERK (SCH772984; SCH), p38 (SB239063; SB) and JNK (SP600125; SP) for 12 h at indicated dose.

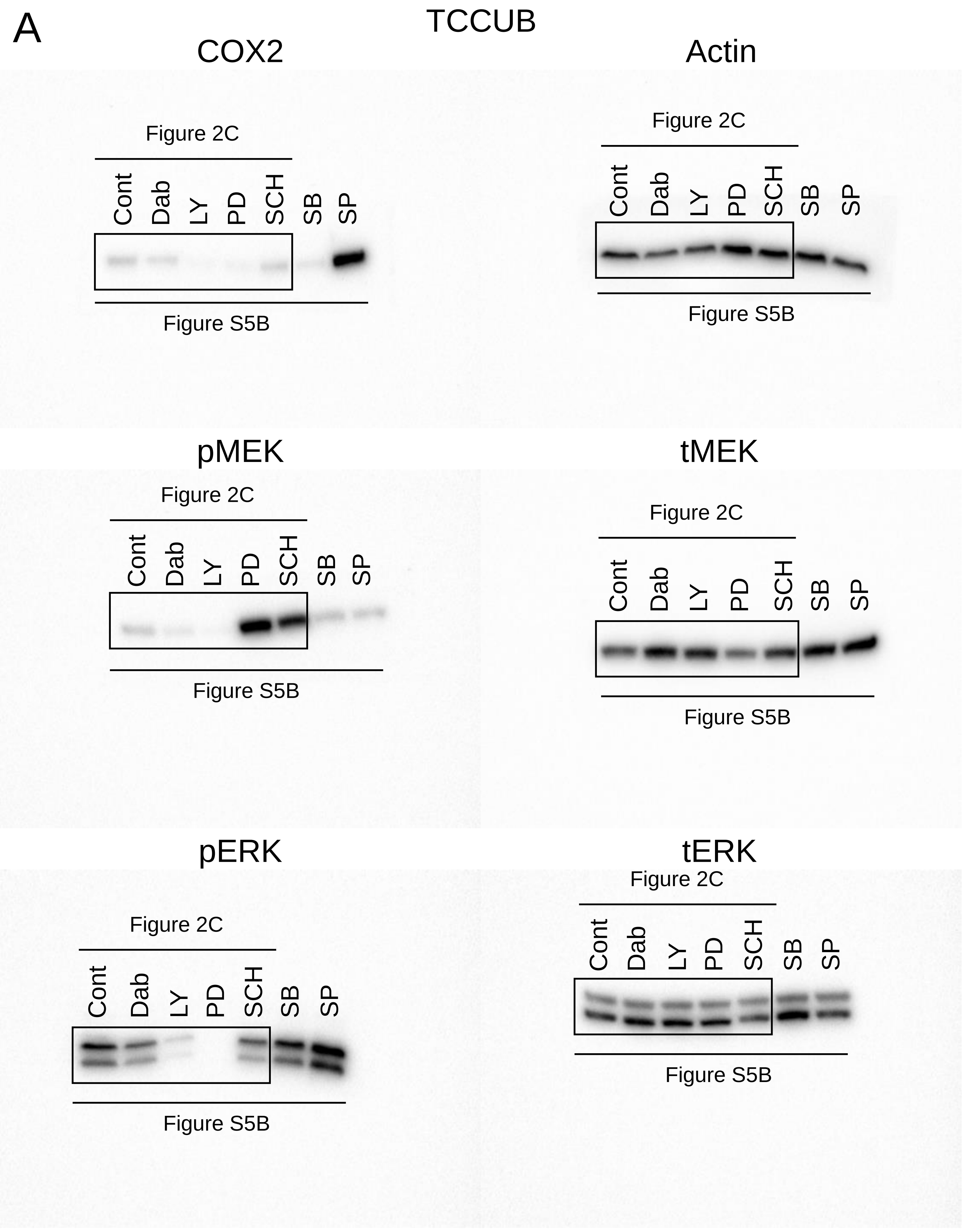
A
TCCUB
COX2
Actin
Figure 2C
Cont
Dab
LY
PD
SCH
SB
SP
Figure S5B
pMEK
tMEK
pERK
tERK

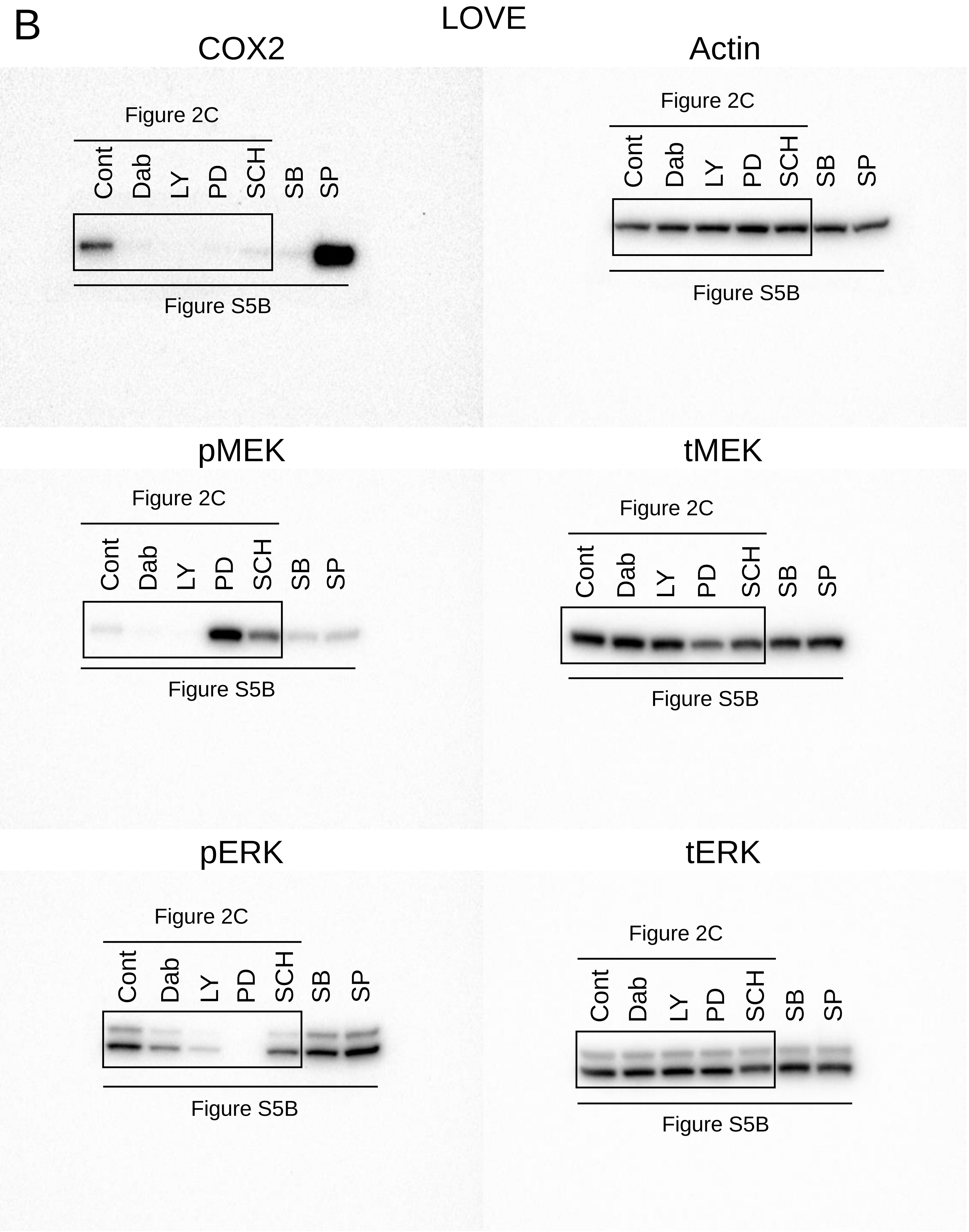
B
LOVE
COX2
Figure 2C
Cont
Dab
LY
PD
SCH
SB
SP
Figure S5B
Actin
Figure 2C
Cont
Dab
LY
PD
SCH
SB
SP
Figure S5B
pMEK
Figure 2C
Cont
Dab
LY
PD
SCH
SB
SP
Figure S5B
tMEK
Figure 2C
Cont
Dab
LY
PD
SCH
SB
SP
Figure S5B
pERK
Figure 2C
Cont
Dab
LY
PD
SCH
SB
SP
Figure S5B
tERK
Figure 2C
Cont
Dab
LY
PD
SCH
SB
SP
Figure S5B

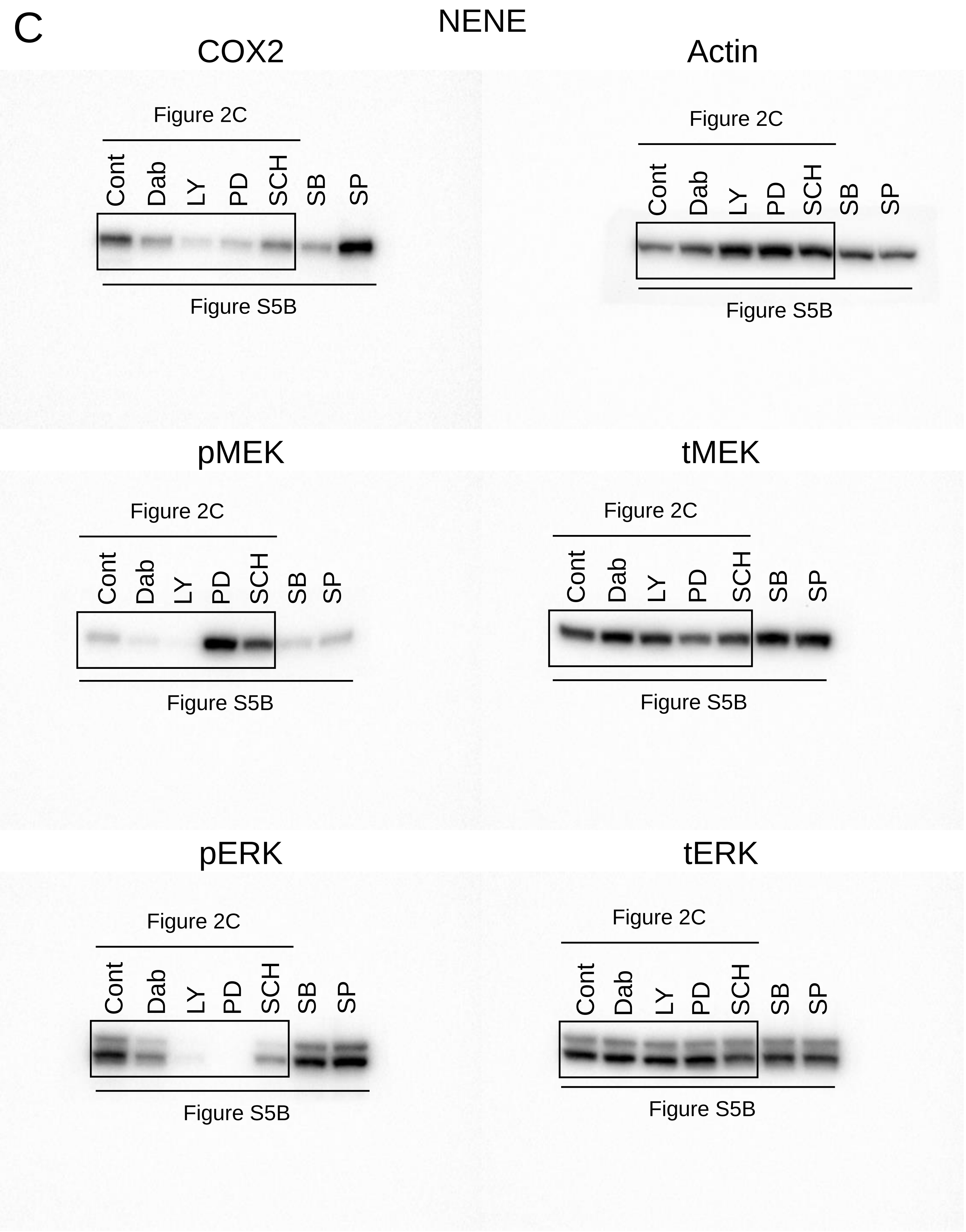
C
NENE
COX2
Figure 2C
Cont
Dab
LY
PD
SCH
SB
SP
Figure S5B
Actin
Figure 2C
Cont
Dab
LY
PD
SCH
SB
SP
Figure S5B
pMEK
Figure 2C
Cont
Dab
LY
PD
SCH
SB
SP
Figure S5B
tMEK
Figure 2C
Cont
Dab
LY
PD
SCH
SB
SP
Figure S5B
pERK
Figure 2C
Cont
Dab
LY
PD
SCH
SB
SP
Figure S5B
tERK
Figure 2C
Cont
Dab
LY
PD
SCH
SB
SP
Figure S5B

D

NMTCC

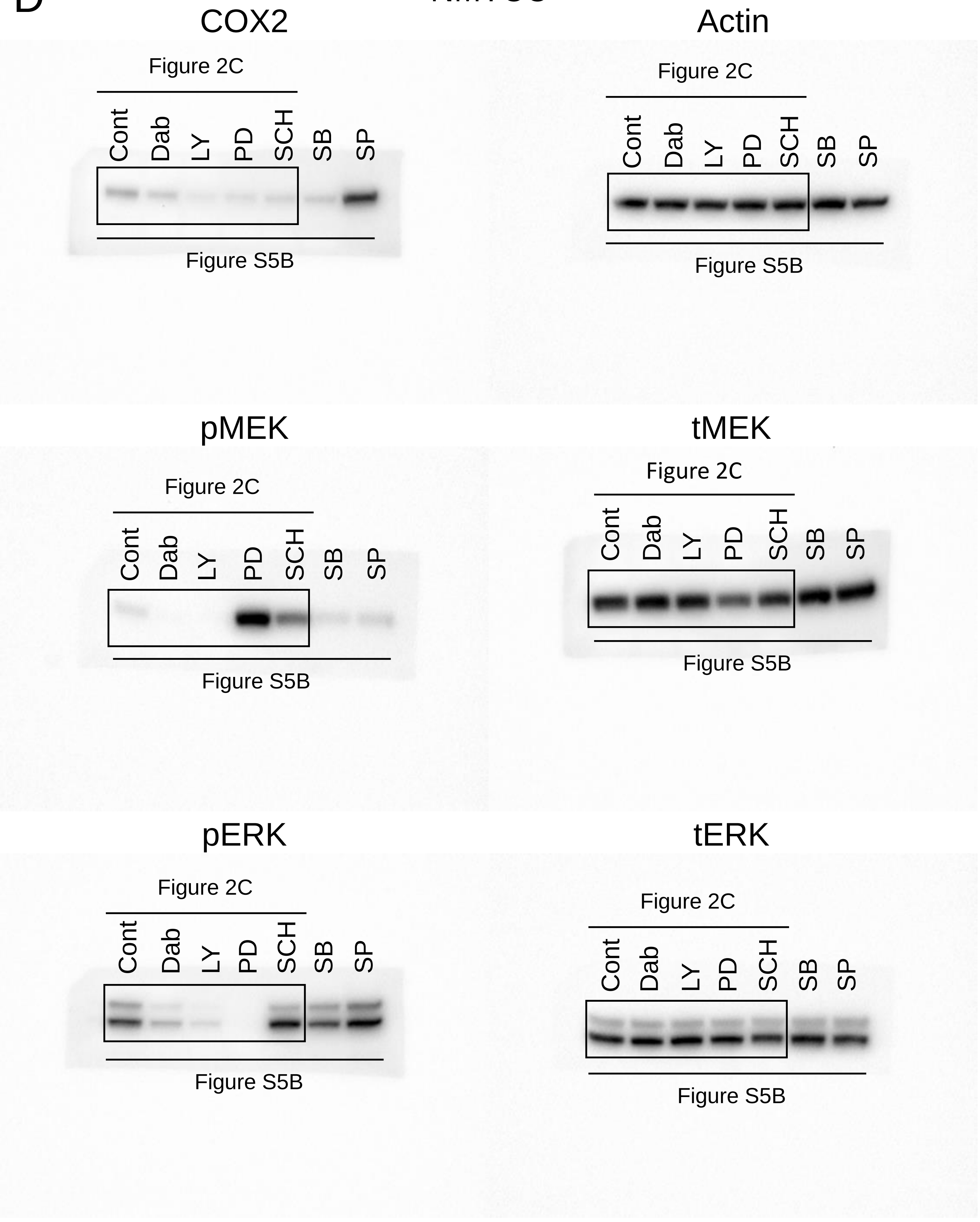
COX2
Figure 2C
Cont
Dab
LY
PD
SCH
SB
SP
Figure S5B
Actin
Figure 2C
Cont
Dab
LY
PD
SCH
SB
SP
Figure S5B
pMEK
Figure 2C
Cont
Dab
LY
PD
SCH
SB
SP
Figure S5B
tMEK
Figure 2C
Cont
Dab
LY
PD
SCH
SB
SP
Figure S5B
pERK
Figure 2C
Cont
Dab
LY
PD
SCH
SB
SP
Figure S5B
tERK
Figure 2C
Cont
Dab
LY
PD
SCH
SB
SP
Figure S5B

E

LTCC

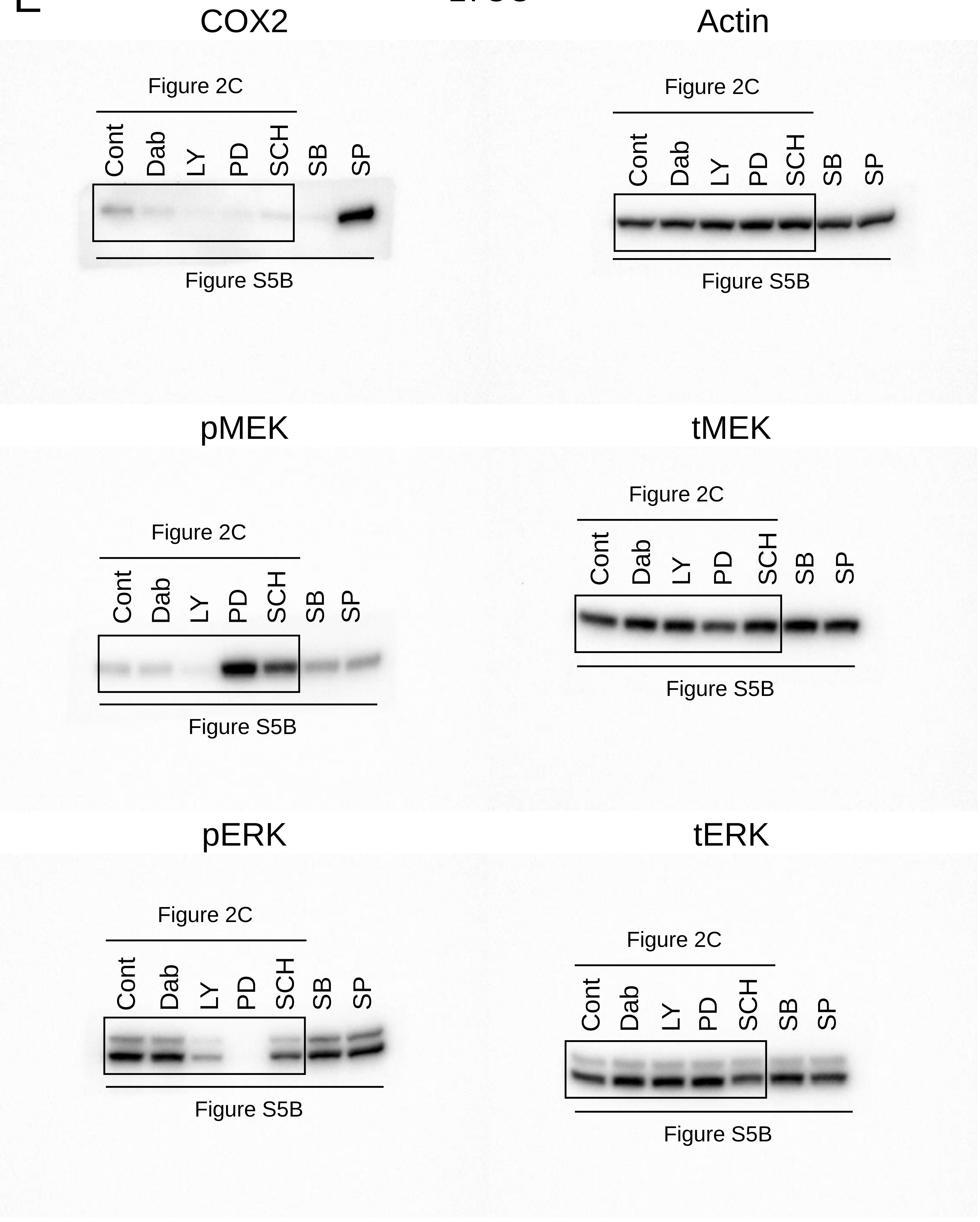

COX2
Figure 2C
Cont
Dab
LY
PD
SCH
SB
SP
Figure S5B
Actin
Figure 2C
Cont
Dab
LY
PD
SCH
SB
SP
Figure S5B
pMEK
Figure 2C
Cont
Dab
LY
PD
SCH
SB
SP
Figure S5B
tMEK
Figure 2C
Cont
Dab
LY
PD
SCH
SB
SP
Figure S5B
pERK
Figure 2C
Cont
Dab
LY
PD
SCH
SB
SP
Figure S5B
tERK
Figure 2C
Cont
Dab
LY
PD
SCH
SB
SP
Figure S5B

F

MCTCC

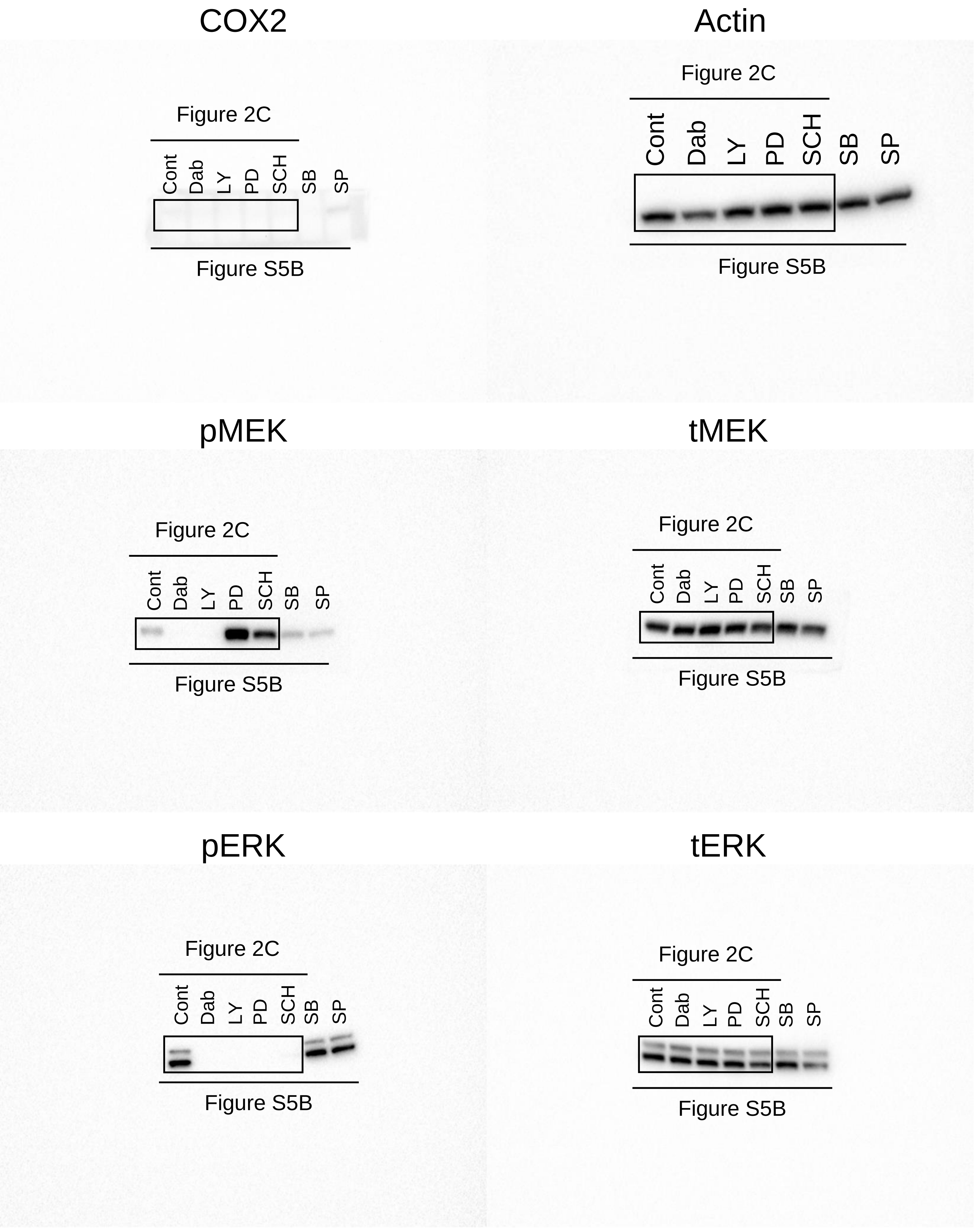
COX2
Figure 2C
Cont
Dab
LY
PD
SCH
SB
SP
Figure S5B
Actin
Figure 2C
Cont
Dab
LY
PD
SCH
SB
SP
Figure S5B
pMEK
Figure 2C
Cont
Dab
LY
PD
SCH
SB
SP
Figure S5B
tMEK
Figure 2C
Cont
Dab
LY
PD
SCH
SB
SP
Figure S5B
pERK
Figure 2C
Cont
Dab
LY
PD
SCH
SB
SP
Figure S5B
tERK
Figure 2C
Cont
Dab
LY
PD
SCH
SB
SP
Figure S5B

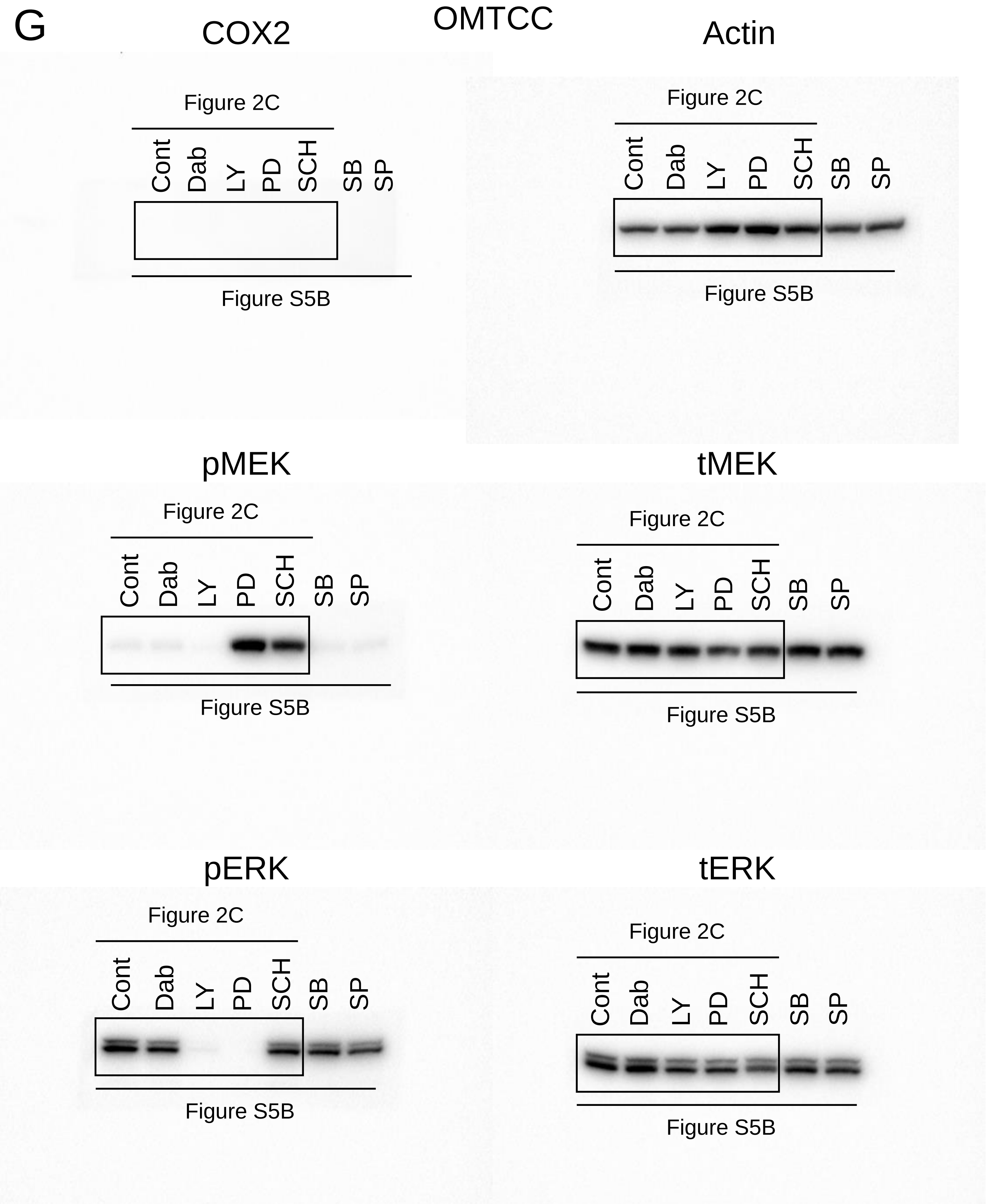

Supplementary Figure S10. Uncropped image of Western blots for Figure 2C and Supplementary Figure S5B. cUC cells were treated with with vehicle (dimethyl sulfoxide; Cont) and inhibitors of BRAF (Dabrafenib; Dab) at 10 µM, pan-RAF (LY3009120; LY) at 1 µM, MEK (PD0325901; PD) at 10 µM, ERK (SCH772984; SCH) at 10 µM, p38 (SB239063; SB) at 10 µM and JNK (SP600125; SP) at 10 µM for 12 h.(A) TCCUB, (B) Love, (C) Nene, (D) NMTCC, (E) LTCC, (F) MCTCC and (G) OMTCC.

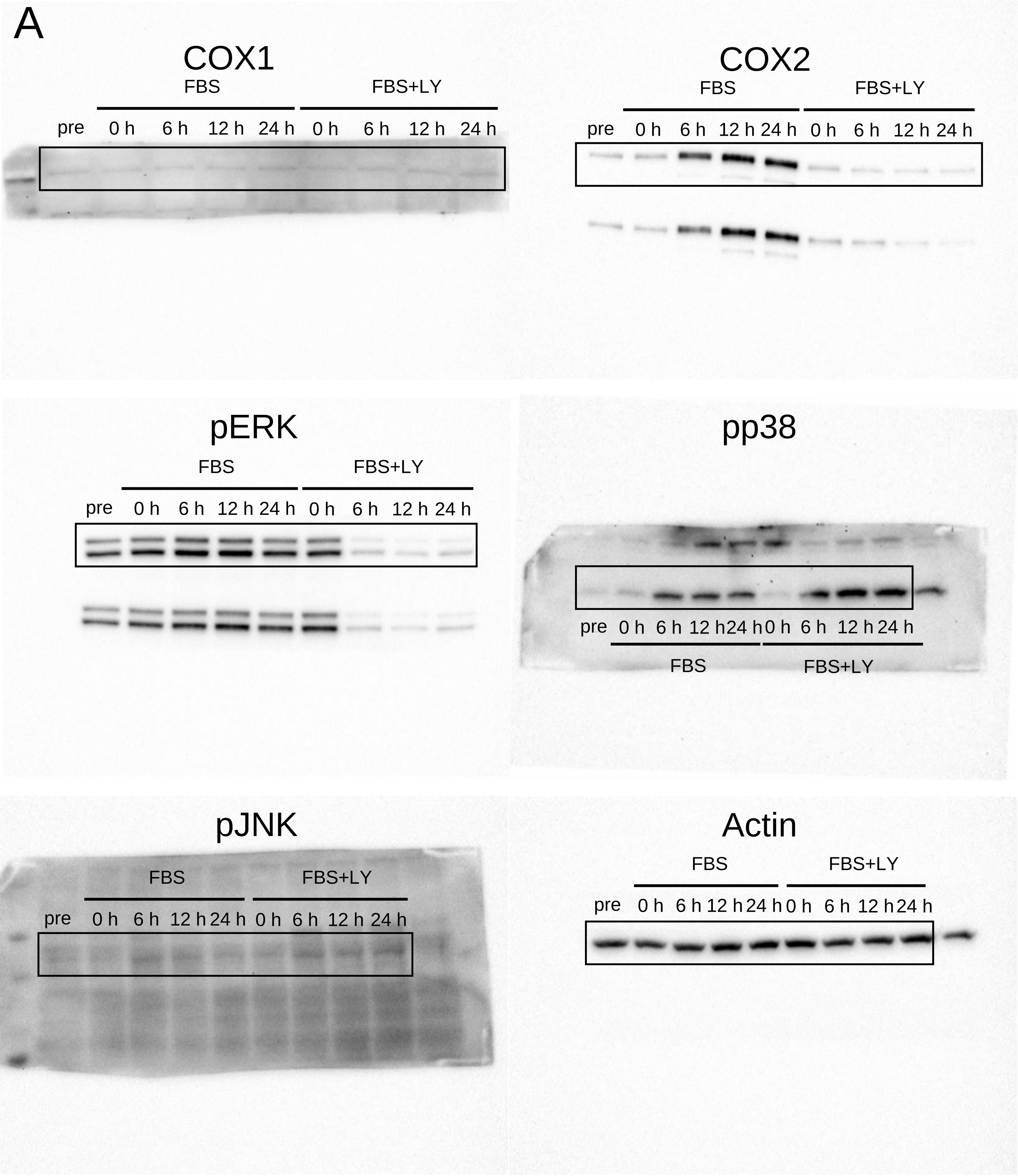
A
COX1
FBS
FBS+LY
pre
0 h
6 h
12 h
24 h
0 h
6 h
12 h
24 h
COX2
FBS
FBS+LY
pre
0 h
6 h
12 h
24 h
0 h
6 h
12 h
24 h
pERK
FBS
FBS+LY
pre
0 h
6 h
12 h
24 h
0 h
6 h
12 h
24 h
pp38
pre
0 h
6 h
12 h
24 h
0 h
6 h
12 h
24 h
FBS
FBS+LY
pJNK
FBS
FBS+LY
pre
0 h
6 h
12 h
24 h
0 h
6 h
12 h
24 h
Actin
FBS
FBS+LY
pre
0 h
6 h
12 h
24 h
0 h
6 h
12 h
24 h

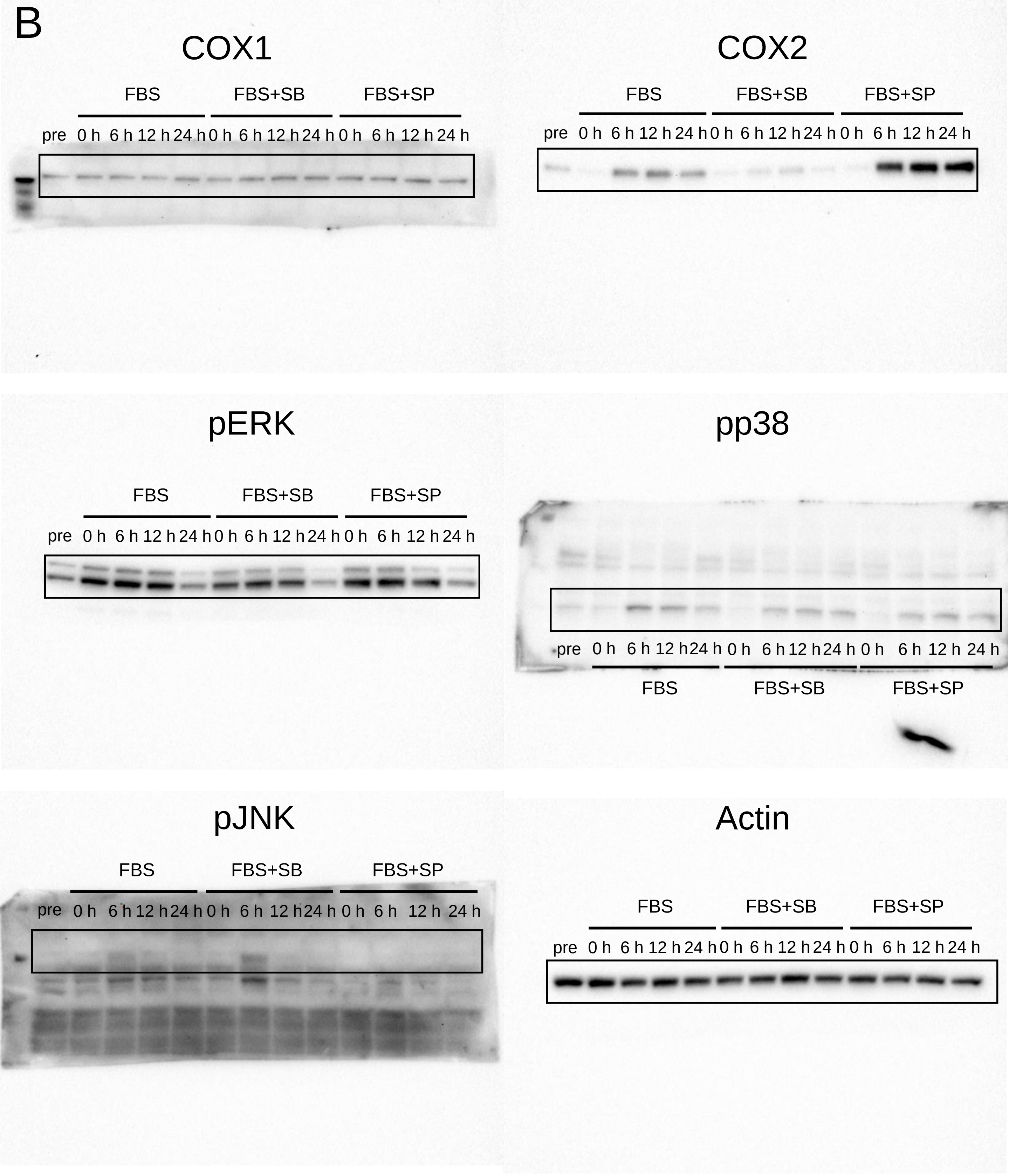

Supplementary Figure S11. Uncropped image of Western blots for Figure 4B. cUC cells (Sora) were treated with foetal bovine serum (FBS) and (A) LY3009120 (LY) at 1 μM, (B) SB239063 (SB) or SP600125 (SP) after 24 h-starvation. Whole cell lysate was corrected before (pre) and after treatment for indicated time.

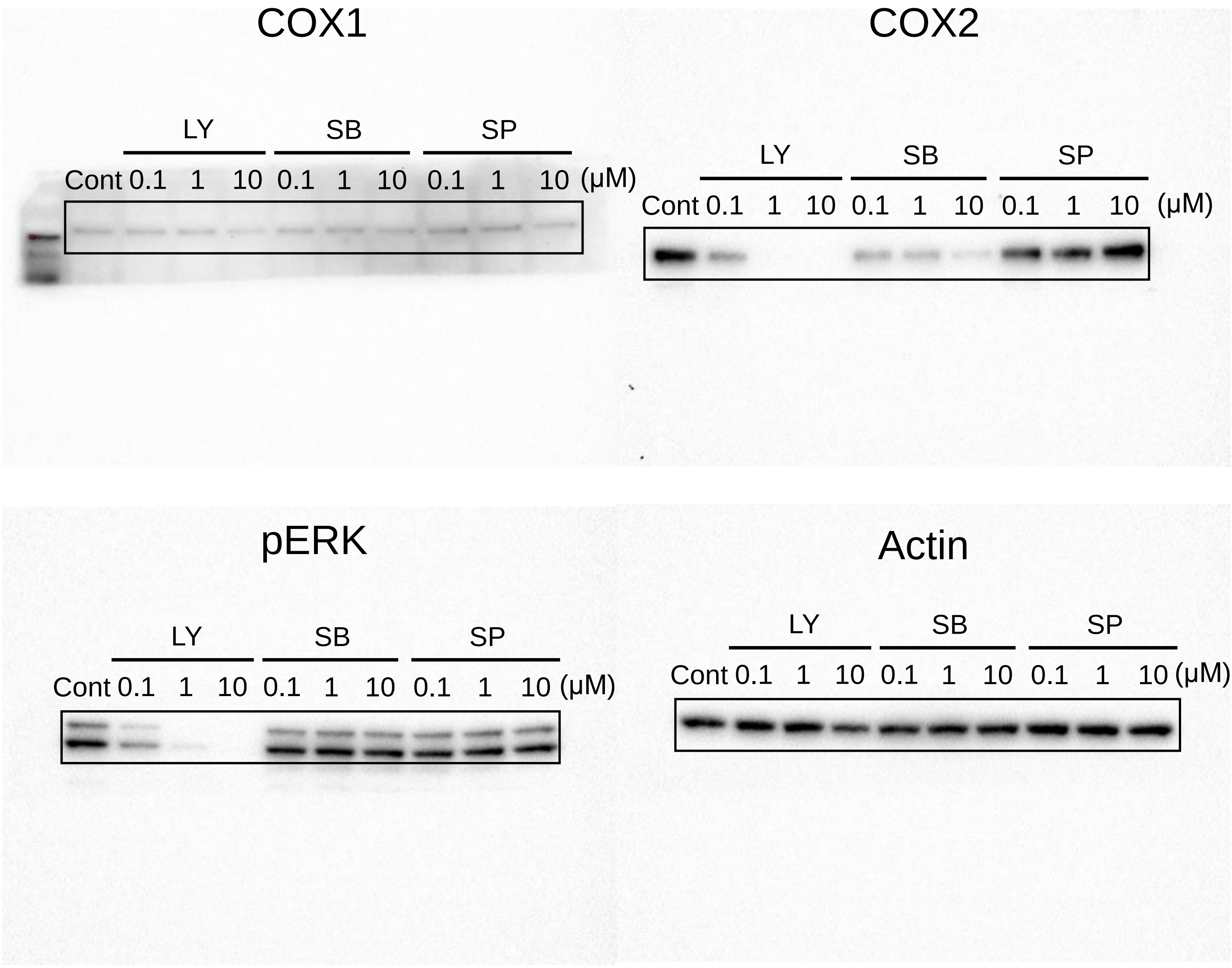

Supplementary Figure S12. Uncropped image of Western blots for Supplementary Figure S6. cUC cells (Sora) was treated with vehicle (dimethyl sulfoxide; Cont) and inhibitors of pan-RAF (LY3009120; LY), p38 (SB239063; SB) and JNK (SP600125; SP) for 12 h at indicated dose.
